# Defining potentially conserved RNA regulons of homologous zinc-finger RNA-binding proteins

**DOI:** 10.1186/gb-2011-12-1-r3

**Published:** 2011-01-13

**Authors:** Tanja Scherrer, Christian Femmer, Ralph Schiess, Ruedi Aebersold, André P Gerber

**Affiliations:** 1 Institute of Pharmaceutical Sciences, Department of Chemistry and Applied Biosciences, ETH Zürich, Wolfgang-Pauli-Strasse 10, 8093 Zürich, Switzerland; 2 PhD Program in Molecular Life Sciences, University and ETH Zürich, Winterthurerstrasse 190, 8057 Zürich, Switzerland; 3 Bioprocess Laboratory, Department of Biosystems Science and Engineering, ETH Zürich, Mattenstrasse 26, 4058 Basel, Switzerland; 4 Institute of Molecular Systems Biology, Department of Biology, ETH Zürich, Wolfgang-Pauli-Strasse 16, 8093 Zürich, Switzerland

## Abstract

**Background:**

Glucose inhibition of gluconeogenic growth suppressor 2 protein (Gis2p) and zinc-finger protein 9 (ZNF9) are conserved yeast and human zinc-finger proteins. The function of yeast Gis2p is unknown, but human ZNF9 has been reported to bind nucleic acids, and mutations in the *ZNF9 *gene cause the neuromuscular disease myotonic dystrophy type 2. To explore the impact of these proteins on RNA regulation, we undertook a systematic analysis of the RNA targets and of the global implications for gene expression.

**Results:**

Hundreds of mRNAs were associated with Gis2p, mainly coding for RNA processing factors, chromatin modifiers and GTPases. Target mRNAs contained stretches of G(A/U)(A/U) trinucleotide repeats located in coding sequences, which are sufficient for binding to both Gis2p and ZNF9, thus implying strong structural conservation. Predicted ZNF9 targets belong to the same functional categories as seen in yeast, indicating functional conservation, which is further supported by complementation of the large cell-size phenotype of *gis2 *mutants with *ZNF9*. We further applied a matched-sample proteome-transcriptome analysis suggesting that Gis2p differentially coordinates expression of RNA regulons, primarily by reducing mRNA and protein levels of genes required for ribosome assembly and by selectively up-regulating protein levels of myosins.

**Conclusions:**

This integrated systematic exploration of RNA targets for homologous RNA-binding proteins indicates an unexpectedly high conservation of the RNA-binding properties and of potential targets, thus predicting conserved RNA regulons. We also predict regulation of muscle-specific genes by ZNF9, adding a potential link to the myotonic dystrophy related phenotypes seen in *ZNF9 *mouse models.

## Background

Post-transcriptional gene regulation is thought to contribute to the limited correlation between mRNA and protein levels in cells [1-4]. A pivotal role for post-transcriptional gene regulation is assigned to RNA-binding proteins (RBPs), which control almost every aspect of a RNA's life, including RNA processing, splicing, export, localization, decay and translation in the cytoplasm [5,6]. An increasing number of studies in diverse model organisms, applying genomic tools such as DNA microarrays or next-generation sequencing, revealed that RBPs specifically bind to distinct RNA groups that often encode functionally related proteins [7-9]. Such organization of RNAs into 'post-transcriptional operons' or 'RNA regulons' may allow the coherent coordination of mRNA fates [5,10,11]. However, how such coordination by RBPs impacts gene expression at the mRNA and protein levels remains largely elusive.

RBPs are often composed of an array of RNA-binding domains that ultimately define RNA-binding specificity. Zinc-finger (ZnF) domains are common, relatively small protein motifs that contain conserved cysteine and histidine residues coordinated to zinc ions. First identified as specific DNA-binding motifs in transcription factors, many different types of ZnF motifs have now been characterized interacting specifically with DNA, RNA, proteins, and lipids [12-14]. Gis2 (Glucose inhibition of gluconeogenic growth suppressor 2) from the yeast *Saccharomyces cerevisiae *is a cytoplasmic protein that contains seven CCHC or 'retroviral'-type ZnF motifs, which are predicted to mediate DNA and possibly RNA interactions. The *GIS2 *gene was originally identified in a screen for multicopy suppressors of the galactose utilization defect of *snf1 mig1 srb8/10/11 *triple mutants, where it genetically interacted with *CDC25*, establishing a functional connection to the Ras/cAMP pathway [15]. In a global screen for mutants with altered cell size, *gis2Δ *mutants were found to display increased cell sizes [16]. Furthermore, an extensive bioinformatics study integrating a variety of microarray-based gene expression profile data suggested that Gis2 is co-regulated with factors contributing to cytoplasmic ribosome function - some of them crucial for cell size control - predicting roles of Gis2p in ribosome biogenesis [17,18].

The human homolog of Gis2p, termed ZNF9 or cellular nucleic acid binding protein (CNBP), contains an additional arginine-glycine-glycine (RGG)-rich motif located between the first and the second of the seven ZnFs (the domain structure and a multiple amino acid sequence alignment is shown in Additional file 1). The protein was first described to bind to purine-rich single-stranded DNA (ssDNA) of the sterol response element, possibly playing a role in sterol metabolism [19]. Several studies further suggested functions of ZNF9 as a positive or negative regulator of transcription by binding to guanosine-rich ssDNA sequences: ZNF9 represses the expression of the beta-myosin heavy chain [20] and the JC virus control region [21], whereas it activates expression of the *c-myc *oncogene [22] and macrophage colony-stimulating factor (M-CSF) [23]. ZNF9 has also been suggested to interact with RNA [24], possibly regulating translation of ribosomal protein mRNA in the frog *Xenopus laevis *[25], or promoting cap-independent translation of ornithine decarboxylase [26,27]. Importantly, ZNF9 has been shown to interact with the 5'-UTR of terminal oligopyrimidine (TOP) tract mRNAs in mammals, and modulates translation efficiency [27].

ZNF9 attracted great interest when Ranum and colleagues [28] found that CCTG nucleotide repeat expansions in the first intron of *ZNF9 *cause myotonic dystrophy type 2 (DM2). DM2 is characterized by heterogeneous, multi-systemic symptoms, including myotonia [29]. The disease is thought to be caused by RNA gain-of-function of the repeats in the spliced-out intron, which accumulate in nuclear foci sequestering members of the muscleblind-like (MBNL) family of RBPs that regulate alternative splicing (reviewed in [30-32]). The depletion of MBNL1 and other RBPs by these RNA repeats leads to abnormal splicing of several messages important for muscle function, establishing the DM2 phenotype. Phenotypic consequences of *ZNF9 *intron repeat expansions are therefore thought to be indirect and disregard a direct role for ZNF9 in pathogenesis. Nevertheless, in mice, *ZNF9 *has substantial impact on proper development, as shown by the embryonic lethality of *ZNF9*^-/- ^embryos, which show severe forebrain truncations [33]. Heterozygous *ZNF9*^+/- ^mice show phenotypes that are related to DM, adding the possibility that ZNF9 may impact on the expression of muscle-specific genes [34]. Whether ZNF9 could partly contribute to the pathogenesis of DM2 is therefore not fully resolved [27,34].

Here, we applied an integrated genomic and proteomic approach to systematically explore the roles of Gis2 and ZNF9 proteins in RNA expression. Using DNA microarrays, we identified the RNAs that are bound by Gis2p in yeast cells. This allowed us to decode a conserved RNA element sufficient for interaction with both Gis2p and ZNF9. Applying a sample-matched proteomics-transcriptomics approach, we further delineated both positive and negative effects on expression of functionally coherent groups of mRNAs, suggesting strong coordinative functions of Gis2p for gene expression. Finally, based on our results, we predict that human ZNF9 may coordinate the expression of ribosomal RNA processing factors and muscle-related genes, which may relate to phenotypes seen in *ZNF9*^+/- ^mice.

## Results

### Gis2p associates with hundreds of mRNAs encoding functionally related proteins

Gis2p has been predicted to bind nucleic acids because it contains seven conserved ZnFs, and related proteins in vertebrates bind to ssDNA or RNA. To validate whether Gis2p binds RNA and to identify potential target RNAs, we performed RNA affinity purifications with carboxy-terminal tandem-affinity purification (TAP)-tagged Gis2p expressed under the control of its native promoter, and we identified the associated RNAs with DNA microarrays (see Materials and methods). Thereby, RNA isolated from extracts (input) and from the purified samples was competitively analyzed with yeast DNA oligo arrays that contained probes for all annotated yeast ORFs and non-coding RNAs, as well as some intergenic regions. In this assay, the ratio of the two RNA populations at a given array element reflects the enrichment of the respective RNA by Gis2p [7,9].

**Figure 1:**
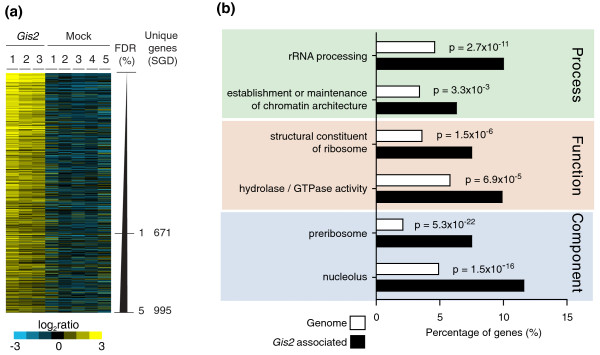
**RNAs specifically associated with yeast Gis2p**. **(a) **Color map of Gis2p-associated transcripts (FDR <5%). Rows represent unique transcripts ordered according to decreasing FDRs. Columns correspond to individual experiments. Relative enrichment of genes in the individual RNA affinity isolations is shown in the blue-yellow ratio scale. **(b) **A selective sample of significantly shared GO terms among Gis2p-associated transcripts with a FDR <5%. SGD, Saccharomyces Genome Database.

To select RNAs that were consistently enriched with Gis2p and hence represent likely targets, we compared the association of transcripts from three independent Gis2p affinity purifications with those from five mock control isolates performed with untagged wild-type cells using Significance Analysis of Microarrays (SAM) [35], and determined false discovery rates (FDRs) for each arrayed feature [8,9]. There were 1,102 features (13.6% of all 8,132 analyzed features), representing 995 transcripts, associated with Gis2p with FDRs of less than 5% (Figure 1a; a list of Gis2p targets is given in Additional file 2; raw data are provided in Additional file 3). Most of these features represented mRNAs (968 exon probes), and we did not see an enrichment of non-coding RNAs, such as rRNAs, tRNAs and small nucleolar RNAs (snoRNAs), suggesting a function of Gis2p on spliced mRNAs in the cytoplasm, which is in agreement with the protein's main localization [36]. However, for 39 of the Gis2p-bound mRNAs, features corresponding to introns or intron-exon junctions were also significantly enriched. Possibly, a minor fraction of Gis2p may associate with some mRNA precursors in the nucleus. We wish to note that some non-target mRNAs may associate with Gis2p and true mRNA targets may dissociate during the affinity-isolation procedure; thus, this assay may not exclusively and completely uncover target mRNAs that are associated with Gis2p *in vivo *[37]. Nevertheless, the identification of a sequence motif among potential Gis2p/ZNF9 targets, the functional links among mRNA targets, and the indications for coordinate expression of functional groups of messages by Gis2p (see below) strongly suggest an underlying biological role for many of the interactions we have identified.

Because many RBPs bind to functionally or cytotopically related mRNAs, we searched for significantly enriched Gene Ontology (GO) terms among the Gis2p-associated messages with AMIGO [38] (a list of significantly enriched GO terms is given in Additional file 4). Indeed, Gis2p was associated with functionally related groups of messages (Figure 1b). Most prominent are messages coding for proteins involved in ribosome biogenesis (*P *< 10^-16^), including the processing of rRNA (*P *< 10^-10^) in the nucleolus (*P *< 10^-15^). Noteworthy, this set of messages contains 7 of the 15 ribosomal cell-size control genes (*P *< 0.01) and regulation of these mRNAs by Gis2p may relate to the enlarged cell sizes of *gis2 *mutant cells [16]. Likewise, Gis2p targets can be clustered to protein complexes acting in rRNA processing/modification, as revealed by analysis for enrichment of protein complexes with FunSpec, an online tool to search for common features among a set of yeast genes [39]. For example, Gis2p targets include the four core protein components of the H/ACA-box snoRNP (Cbf5, Gar1, Nhp2, and Nop10), which catalyzes site-specific pseudouridylation in pre-rRNA, and eight (Nop1, Lcp55, Mpp10, Sof1, Sik1, Imp4, Nop58, Rrp9) out of nine proteins (*P *< 10^-6^) involved in ribose methylation of rRNAs, which is carried out by specific snoRNPs that contain snoRNAs of the C/D-box family [40].

We also found linkage of Gis2p targets with factors that are involved in regulation of chromosome organization or that encode enzymes with hydrolytic activity. In particular, Gis2p was associated with 21 of 55 (38%, *P *< 2 × 10^-5^) messages encoding GTPases that regulate either protein localization or activity, including 11 GTPases that participate in vesicle-mediated transport (Arl1, Arf1, Arf2, Rhb1, Sec4, Vps21, Ypt1, Ypt6, Ypt31, Ypt32, and Ypt52). Interestingly, three of the GTPases are Ras homologs (Ras2, Rho2, and Rsr1), which possibly relate to previous findings that connected Gis2 to Ras signaling [15].

Since the application of an arbitrarily chosen cutoff to select genes may not detect weak associations with functional groupings, we additionally analyzed the entire SAM-score ranked data using the GO enRIchment anaLysis and visuaLizAtion tool (GOrilla), an online tool to define significantly enriched GO terms in a ranked list of data (Additional file 4) [41]. Besides the above-mentioned GO terms, additional GOs for DNA-related processes in the nucleus, such as DNA recombination (*P *< 3 × 10^-5^), transcription (*P *< 9 × 10^-5^) or chromatin modification (*P *< 3 × 10^-4^) were generally enriched, possibly reflecting weaker interaction of respective messages with Gis2p.

### Gis2p binds to GAN repeats within coding sequence of target mRNAs

**Figure 2:**
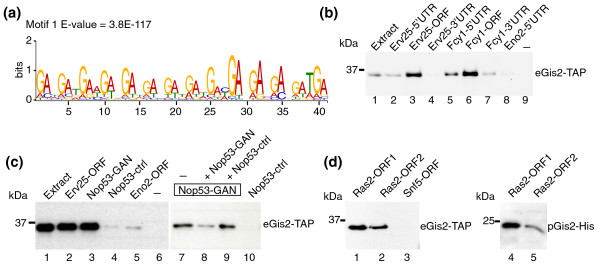
**Gis2p preferentially binds to coding sequences that bear GAN repeats**. **(a) **Conserved sequence element in the ORF of Gis2p targets identified with MEME. The E-value reflects the probability to detect the motif by chance. **(b) **RNA-protein complexes formed between biotinylated RNA fragments and Gis2-TAP were purified on streptavidin beads and monitored by immunoblot analysis. Representative experiments from at least three biological replicates are shown. Biotin labeled fragments comprising the 5'-UTRs (lanes 2 and 5), ORFs (lanes 3 and 6), and 3'-UTRs (lanes 4 and 7) of Erv25 and Fcy1 were incubated with extracts of Gis2-TAP expressing cells (lane 1). Eno2-5'UTR (lane 8) is a negative control RNA derived from the 'non-target' *ENO2 *(lane 8) and a sample without RNA (lane 9) was used to control for RNA-independent binding to the beads. **(c) **RNA pull-downs with RNA fragments derived from *NOP53*. Nop53-GAN (lanes 3 and 7 to 9) contains a GAN-rich sequence element whereas the similarly sized fragment Nop53-ctrl does not (lanes 4 and 10). Erv25-ORF (lane 2) and Eno2-ORF (lane 5) are positive and negative control RNAs, respectively. Binding of Gis2-TAP to Nop53-GAN was competed with a ten-fold excess of non-biotinylated Nop53-GAN (lane 8) but not with excess of Nop53-ctrl (lane 9). **(d) **RNA pull-downs with two fragments derived from the *RAS2 *ORF. Biotinylated RNAs were incubated with extracts from yeast cells expressing Gis2-TAP (eGis2-TAP, lanes 1 to 3) or with Gis2-His expressed and purified from *Escherichia coli *(pGis2-His, lanes 4 and 5). A fragment derived from the ORF of *SNF5 *was used as a negative control RNA (lane 3).

We next wondered whether there are common structural features within mRNA targets that could specify Gis2p interaction. We therefore retrieved the coding sequences (CDSs) of ORFs from the *Saccharomyces *Genome Database (SGD) [42], as well as 3'- and 5'-UTR sequences [43] for the 50 highest scored Gis2p mRNA targets, and we searched for common motifs using Multiple Expectation Maximization for Motif Elicitation (MEME) as an unbiased motif discovery tool [44] (see Materials and methods). MEME identified a consensus sequence composed of 14 GAN trinucleotide repeats (N refers to any of the four nucleotides) within ORFs (median approximately 7 GAN repeats) (Figure 2a). No motif was found among the 3'- and 5'-UTR sequences [43] or when searching 500 bp downstream or upstream of these ORFs covering UTRs (data not shown). Furthermore, GAN repeats are overrepresented in the ORFs of our experimentally defined Gis2p targets with FDR <5% (for example, 98 Gis2p targets among all genomically encoded 232 ORFs that bear at least one (GAN)_7 _sequence element; *P *< 10^-22^), and ORFs with greater numbers of GAN repeats tend to be more highly enriched in Gis2 affinity isolations (the distribution of (GAN)_7_-containing ORFs in Gis2-TAP affinity isolations is shown in Additional file 5). These results let us propose that Gis2p may bind to stretches of GAN repeats, which are preferentially located in ORFs/CDSs of mRNA targets.

To validate some of our experimentally determined mRNA targets and the *in silico *predicted RNA recognition motif, we performed a series of RNA pull-down experiments with biotinylated RNAs added to extracts derived from Gis2-TAP cells or with recombinant yeast Gis2p and human ZNF9 (see Materials and methods). We tested RNAs derived from four of our experimentally determined Gis2p targets (Fcy1, Erv25, Nop53, Ras2; Gis2p association with FDRs approximately 0%). We synthesized RNA fragments covering the ORF, approximately 500 nucleotides upstream, and approximately 500 nucleotides downstream of the sequences of *FCY1 *and *ERV25*. Gis2-TAP best interacted with RNA fragments derived from the ORFs, and substantially weaker interactions were seen with RNA fragments covering the 5'-UTR (upstream region), and faint (*FCY1*) or no interaction (*ERV25*) with downstream fragments including 3'-UTRs. Thus, tagged Gis2p interacts preferentially via RNA elements located in the ORFs of these messages (Figure 2b). To test for potential involvement of GAN repeats within the natural context, we further analyzed the interactions of Gis2p with fragments derived from ORFs of *NOP5 *and *RAS2*. As expected, Gis2p bound efficiently to a transcript encompassing the sequence (GAA)_7 _(Nop53-GAN), but it did not interact with a similarly sized control fragment that lacks GAN repeats (Nop53-ctrl) (Figure 2c). Moreover, addition of a ten-molar excess of Nop53-GAN RNAs to the assay strongly prevented binding of Gis2p to biotinylated RNAs, but no competition was seen with the Nop53-ctrl RNA (Figure 2c). Likewise, two fragments derived from the *RAS2 *CDS, both containing a (GAN)_3 _sequence, efficiently pulled-down tagged Gis2p that was either derived from yeast (eGis2-TAP) or heterologously expressed and purified from *Escherichia coli *(pGis2-His; Figure 2d). Noteworthy, we repeatedly observed that Gis2p was approximately two-fold more efficiently recovered with one of the fragments (Ras2-ORF1), possibly due to the presence of a (GAN)_2_NNNGA sequence located 39 nucleotides upstream of a (GAN)_3 _sequence. In conclusion, these results confirm specific interactions of Gis2p with four of our experimentally defined mRNA targets, and further indicate that Gis2p preferentially associates with RNA elements that enclose GAN repeats in CDSs. To our knowledge, Gis2 is therefore the first ZnF protein known to bind to CDSs.

### Conserved RNA and DNA binding specificities of yeast Gis2p and human ZNF9

**Figure 3:**
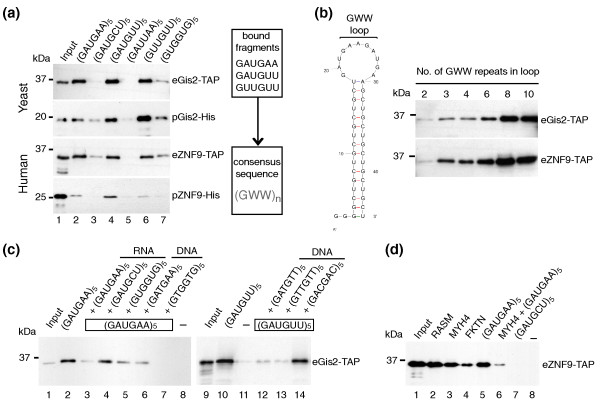
**Gis2p and ZNF9 bind specifically to GWW repeats in RNA and to G-rich sequences in ssDNA**. RNA-protein complexes formed between biotinylated RNAs and yeast extracts expressing Gis2-TAP or ZNF9-TAP (eGis2/eZNF9) or recombinant Gis2-His or ZNF9-His purified from *E. coli *(pGis2/pZNF9) were captured with streptavidin beads and visualized by immunoblot analysis with specific antibodies detecting the TAP or His tag. Representative experiments from at least three biological replicates are shown. **(a) **RNA pull-downs with short biotinylated RNAs bearing different nucleotide triplet repeats (lanes 2 to 7). The consensus sequence for protein-RNA interaction is depicted on the right. **(b) **Testing different sizes of GWW loops for interaction with Gis2p/ZNF9 (lanes 1 to 6). The predicted stem-loop structure with varying sizes of GWW-loops is shown to the left. **(c) **RNA pull-downs after the addition of ten-fold excess of non-labeled competitor RNA (lanes 3 to 5) or ssDNA (lanes 6, 7, and 12 to 14). No RNA was added to control for unspecific binding of proteins to the beads (lanes 8 and 11). **(d) **Binding ZNF9 to human RNAs containing at least three GWW repeats in the coding region (lanes 2 to 4). (GAUGAA)_5 _was used as positive control (lane 5), and (GAUGCU)_5 _as negative control (lane 7). Binding of ZNF9 to *MYH4 *RNA was efficiently competed with ten-fold excess of unlabeled (GAUGAA)_5 _RNA (lane 6). A reaction without RNA is shown in lane 8.

Since yeast Gis2p and human ZNF9 are well conserved across the seven ZnFs, we wondered whether the respective RNA binding preferences are conserved as well. To examine this idea and to gain a more detailed insight into the RNA-binding specificities of the yeast and human protein, we tested a series of short 30-mer RNA oligonucleotides, comprising ten trinucleotide (triplet) repeats in RNA pull-down assays. We therefore incubated the RNAs with yeast extracts containing either tagged protein (eGis2-TAP, eZNF9-TAP) or with partially purified proteins expressed in *E. coli *(pGis2-His, pZNF9-His; see Materials and methods) to evaluate whether the observed interactions are direct. Biotinylated (GAUGAA)_5 _efficiently pulled-down Gis2p as well as ZNF9, which is in agreement with our postulated necessity of GAN repeats for protein interaction (Figure 3a). Thereby, guanosine at the first position of the triplet is essential for interaction as no binding was seen with short RNAs in which half of the Gs were changed to uridine (GAUUAA)_5_. We further analyzed binding selectivity for the second and third position of the triplet repeats: RNA oligos (GAUGUU)_5 _and (GUUGUU)_5_, in which adenosines at the second and/or third position were changed to uridine, still bound Gis2p and ZNF9. However, changing the adenosines at the second position to cytosine (GAUGCU)_5 _or to guanosine (GUGGUG)_5 _almost completely abrogated binding. Likewise, we observed substantially weaker binding to (GUGGUG)_5 _and (GACGAC)_5_, where the third position of the triplet was changed to guanosine and cytosine, respectively (Figure 3a, and data not shown). In conclusion, these experiments demonstrate that both the yeast and human protein bind specifically to GWW repeats (W = A/U). Noteworthy, this consensus is somewhat different to the GAN repeats enriched among the experimentally defined Gis2p targets. A search among all yeast ORFs for the presence of (GUN)_3 _trinucleotide repeat sequences - which includes the potential GUA and GUU binding sites for Gis2p - revealed that these sequences are simply not present among yeast ORFs. Thus, although Gis2p has broader specificity for GWW repeats, it can only associate with GA(A/U) repeats in yeast ORFs because of restrictions set by the genome.

To test how many (GWW) repeats are optimal for Gis2p and ZNF9 binding, we further designed RNA probes with various numbers of GWW repeats flanked by (GCU)_5 _sequences. This design was imposed to form a defined stem-loop secondary structure displaying the GWW repeats in the unstructured loop as single stranded RNA, although some steric hindrances may be caused by the artificial stem (Figure 3b). We found that association of both Gis2p and ZNF9 with RNA gradually increased with the number of GWW repeats: three GWW repeats were minimally required to detect robust binding, best binding was seen with six to eight repeats, and more than eight repeats did not further enhance binding (Figure 3b). Since these proteins harbor seven ZnFs, we speculate that in this case every ZnF interacts with one GWW repeat, but further experiments are required to define binding sites in the protein.

ZNF9 has been reported to bind G-rich sequences in ssDNA [24], and we investigated whether Gis2p binds to ssDNA as well. We therefore performed competition assays with RNA or ssDNA (Figure 3c). In agreement with our previous results, the binding of Gis2p to biotinylated (GAUGAA)_5 _RNA was strongly reduced by the addition of excess unmodified (GAUGAA)_5 _competitor, but it was only weakly to moderately diminished by addition of (GAUGCU)_5 _and (GUGGUG)_5_. Interestingly, we reproducibly observed opposite outcomes when competing with ssDNA. The GWW repeat bearing DNA fragments (GATGAA)_5_, (GATGTT)_5, _and (GTTGTT)_5 _moderately competed for Gis2p binding, but strong competition was seen with the G-rich oligonucleotide (GTGGTG)_5 _(Figure 3c). Likewise, direct DNA pull-down experiments with biotinylated (GTGGTG)_5 _DNA revealed strong association with Gis2p (data not shown). Thus, these results suggest that Gis2p employs different selectivity for RNA and DNA, the latter depending on G-rich sequences [24], which may pinpoint specific roles for these proteins in DNA and RNA regulation.

### GWW repeats occur mostly at the first codon position in CDSs of Gis2p targets

We examined the distribution of GWW repeats of various lengths in UTRs and ORFs among all annotated transcripts and among our experimentally defined list of Gis2p mRNA targets. GWW stretches occur most frequently in ORFs (for example, 1,057 transcripts with [GWW]_4_), which are significantly enriched among our experimentally defined set of Gis2p targets (235 transcripts) (Table 1). The same analysis with (G[C/G][C/G])_4 _repeats, a related motif that is not bound by Gis2p, revealed only six ORFs that bear this sequence, none of them being a Gis2p target. The GWW repeats are much rarer in UTRs: only 55 transcripts have a (GWW)_4 _motif in the 3'-UTR, among them 22 Gis2p targets (*P *< 10^-5^). This analysis indicates that although GWW repeats occur in different regions of transcripts, most of them are in CDSs representing likely binding sites for Gis2p. Thereby, the motifs are preferentially positioned in-frame (first codon position), which has consequences for the amino acid composition of the encoded proteins: 1,309 (89%) of the 1,438 (GWW)_4 _motifs encoded in the CDS genome, as well as 416 (93%) of 446 the motifs present in Gis2p targets, start at the first codon position. Moreover, since GA(A/U) codons specify the polar acidic amino acids glutamate (GAA) and aspartate (GAU), it is not further surprising that messages for proteins that contain stretches of these amino acids are preferentially targeted by Gis2p (for example, 93 of 229 (D/E)_7_-containing proteins are among our experimentally defined Gis2p targets; *P *< 3 × 10^-14^). A similar bias has recently been seen for Khd1p, which preferentially binds to CNN repeats that are positioned in-frame in CDSs [45]. Whether the preference for certain RBPs to interact with short triplet repeats positioned in-frame in CDSs has some functional implications has to be further investigated, but it may indicate some functional link to translation.

**Table 1:** Number of GWW motifs found in all transcripts and among Gis2p targets

Motif	Location	**Number of motifs**^**a**^	Number of Gis2 targets	***P*-value**^**b**^
(GWW)_3_	ORF	3,550	606	0.7
	3'-UTR	347	77	0.01
	5'-UTR	191	26	0.24
				
(GWW)_4_	ORF	1,057	235	2 × 10^-8^
	3'-UTR	55	22	4 × 10^-5^
	5'-UTR	20	3	1
				
(GWW)_5_	ORF	280	106	1 × 10^-17^
	3'-UTR	10	1	1
	5'-UTR	1		1
				
(GWW)_6_	ORF	106	55	9 × 10^-17^
	3'-UTR	4		
	5'-UTR	1		
				
(GWW)_7_	ORF	61	34	5 × 10^-12^
	3'-UTR	4		
				
(GWW)_8_	ORF	35	20	8 × 10^-8^
	3'-UTR	3		

^a^ORFs from SGD (5,885 genes), UTR sequences from [43]. ^b^Probability that motifs are enriched in Gis2p targets by chance (Fisher's test).

Despite these correlations with GWW repeats, we wish to note that as we did not detect the cognate consensus sequence elements in all the experimentally identified targets, alternative sequences or structural elements in the RNA might also allow specific interactions with Gis2p; for instance, the repeats could be dispersed in the transcript (for example, *RAS2*). Some mRNAs may also have been associated indirectly as part of larger complexes.

### Conservation of functional groups among yeast and predicted human mRNA targets

**Figure 4:**
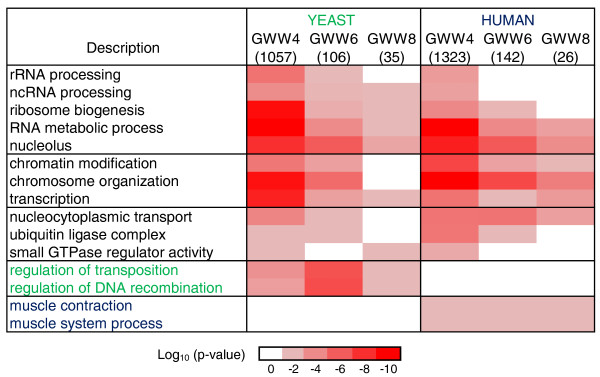
**Significantly shared GO terms among predicted Gis2p and ZNF9 mRNA targets**. GO term enrichments were assessed for predicted mRNA targets with GWW repeats of different lengths in CDSs. Numbers in brackets indicate the total number of genes in the respective group for which GO annotations were available. The color intensity corresponds to the log_10 _*P*-value. GO terms labeled in green were only enriched in yeast, the ones in blue only in human. ncRNA, non-coding RNA.

The identification of a defined recognition motif in the RNA for these ZnF proteins allows the prediction of human mRNA targets of ZNF9. We retrieved annotations for all yeast and human protein coding genes that bear GWW repeats of various lengths within their CDSs (a list of these yeast and human genes is provided in Additional files 6 and 7, respectively), and we searched for significantly enriched GO terms among them with AMIGO. We found a remarkable coincidence of functional classifications of the proteins encoded by the human and yeast messages, such as ribosome biogenesis, chromatin modification, and GTPase mediated signaling pathways (Figure 4). Most of these themes were also seen among the experimentally determined Gis2p targets, further substantiating the predictive power of our analysis (Figure 1b). Functionally related groups that were exclusively present in either organism mainly relied on the absence of homologous proteins. Foremost, we were intrigued by the significant enrichment of transcripts of genes that code for proteins acting in 'muscle contraction' and 'muscle system process' among the predicted ZNF9 targets. This includes nine genes coding for microfilament motors (*MYH2*, *MYH3*, *MYH4*, *MYH7*, *MYH9*, *MYH10*, *MYH13*, *MYO3A*, *MYO6*; *P *< 2.5 × 10^-6^), four genes encoding proteins of the troponin complex (*TNNT2*, *TNNT3*, *TNNI1*, *TNNI2*; *P *< 5.4 × 10^-3^) and five genes for voltage-gated sodium channels (*SCN3A*, *SCN4A*, *SCN7A*, *SCN9A*, *SCN11A*; *P *< 2 × 10^-3^), which are required for action potential production and propagation in muscle cells and hence are a prerequisite for muscle contraction [46].

We further tested the binding of ZNF9 to predicted human targets encoding muscle proteins by RNA pull-down assays (Figure 3d). We examined transcripts encompassing the CDSs for M-RAS, encoding a muscle-specific RAS protein; the myosin heavy chain protein MYH4, which is primarily expressed in skeletal muscles; and fukutin (FKTN), the locus for Fukuyama congenital muscular dystrophy. ZNF9 selectively interacted with all of those RNA substrates, likely via four (*MYH4*, *FKTN*) or five (*M-RAS*) consecutive GWW repeats located in CDSs (Figure 3d).

### Conserved impact of Gis2 and ZNF9 on cell size and growth

**Figure 5:**
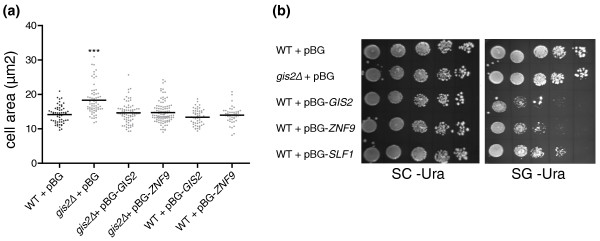
**Phenotypic analysis of yeast cells**. **(a) **Cell-size analysis. Wild-type (WT) and *gis2Δ *cells harboring either the empty vector (+ pBG) or galactose-inducible *GIS2 *or *ZNF9 *were grown in synthetic medium supplemented with 2% galactose to late mid-log phase. The cell area relating to the cell-size is depicted for 50 cells and the average is marked with a black line (***P < 0.0001). **(b) **Vegetative growth. WT cells or *gis2Δ *cells harboring either the empty vector (+ pBG) or galactose-inducible *GIS2*, *ZNF9 *or *SLF1 *(positive control for vegetative growth defect) were serially diluted (1:5) and spotted on the indicated plates: SC -Ura, synthetic complete medium lacking uracil; SG -Ura, the same as SC -Ura but also containing galactose instead of 2% glucose.

Puzzled by the finding that functionally related groups such as rRNA biogenesis components and components of the Ras signaling pathway were particularly shared between the yeast Gis2p and predicted human ZNF9 mRNA targets, we wondered whether the human ZNF9 protein could complement the large cell-size phenotype of *gis2Δ *mutant cells [16]. We therefore compared the cell sizes of *gis2Δ *mutant and wild-type cells with *gis2Δ *cells that overexpress *GIS2 *or *ZNF9 *on a plasmid (Figure 5a). Whereas *gis2Δ *cells showed a significantly larger size compared to the control (*P *= 0.0001), we found that *gis2Δ *cells overexpressing *GIS2 *or *ZNF9 *were of similar sized to wild-type cells, showing that both orthologs can fully rescue the *gis2Δ *phenotype. Likewise, we found that the previously reported slight vegetative growth defect of *GIS2 *overexpressing cells could be fully recapitulated by *ZNF9 *overexpression [47] (Figure 5b). Thus, *ZNF9 *can complement *GIS2*-dependent phenotypes in yeast, suggesting functional conservation between homologous proteins. This is in accordance with our finding that both proteins recognize identical elements in mRNAs coding for similar functional classes of proteins.

### Gis2p selectively represses expression of ribosome biogenesis factors and increases expression of motor proteins

To analyze the global impact of Gis2p on gene expression, we compared mRNA levels of *gis2Δ *cells with wild-type control cells grown to mid-log phase using DNA microarrays (normalized microarray data are provided in Additional file 8); 227 genes representing 4% of all analyzed features changed more than 1.5-fold with *P *< 0.05 (one sample *t*-test), indicating that deletion of *GIS2 *does not drastically affect global mRNA expression (Venn diagrams displaying the fraction of up- or down-regulated genes are shown in Additional file 9). Among the 190 up-regulated features are 40 (21%) Gis2p targets, which are thus slightly overrepresented (*P *= 0.05, Chi-square test). A search for common functional attributes among the differentially regulated genes indicate some overrepresentation of ribosomal proteins among the up-regulated ones (15 genes, *P *< 0.008), and 9 out of 36 down-regulated genes code for proteins that act in pheromone response (*P *< 2.2 × 10^-8^) (a list of significantly enriched GO terms is given in Additional file 8).

**Figure 6:**
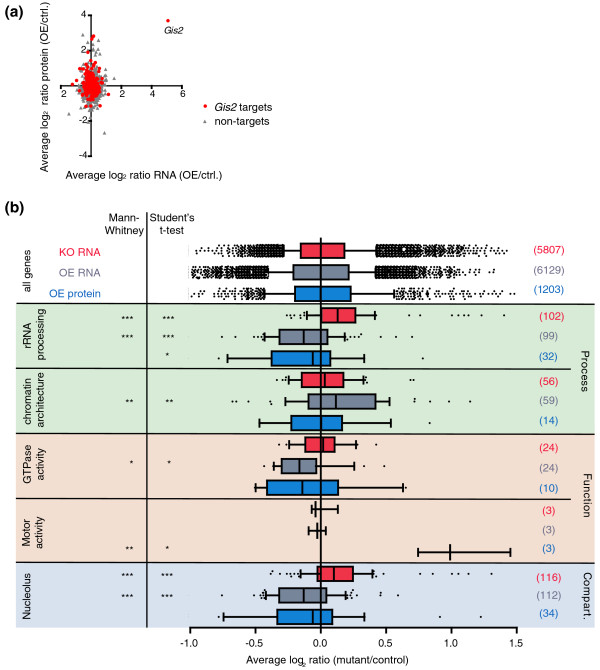
**Transcriptome and proteome expression analysis of Gis2p targets**. **(a) **Scatterplot depicting relative changes of protein levels (*y*-axis) compared to mRNA levels (*x*-axis). Gis2p targets are shown in red, non-targets in grey. **(b) **Boxplots depicting relative changes of mRNA levels in *gis2Δ *cells compared to wild-type cells (knockout (KO) RNA; colored in red), and mRNA (overexpressing (OE) RNA; grey) and protein (OE protein; blue) levels of cells overexpressing *GIS2*. Whiskers extend from the 10th to the 90th percentile. The distribution of all features is shown on top (all genes). Genes annotated to the GO terms indicated to the left were retrieved from the SGD, and the number of plotted Gis2p target mRNAs/proteins within the respective GO group is indicated in brackets. Asterisks refer to *P*-values determined in a Mann-Whitney test and Student's *t*-test with Welch's correction comparing the distribution of Gis2p target genes assigned to specified GO term with the distribution of all measured features: ***P < 0.001; **P < 0.01; *P < 0.05.

We also performed the opposing experiment and overexpressed *GIS2*. Therefore, yeast cells harboring a plasmid with *GIS2 *under the control of a galactose-inducible promoter, and control cells containing an empty plasmid, were grown to mid-log phase, and expression was induced for 1.5 hours with 2% galactose. We reasoned that inducible short-time overexpression may be beneficial to minimize secondary effects that may occur after prolonged changes in Gis2 expression levels, as possibly seen in *gis2Δ *cells. The relative changes in mRNA and protein levels of *GIS2*-overexpressing compared to control cells were then measured with DNA microarrays and quantitative mass spectrometry (qMS), respectively. We obtained mRNA data for 6,129 genes and quantitative proteomics data for 1,203 different proteins (raw data from the microarray and qMS analysis upon *GIS2 *overexpression are provided in Additional files 10 and 11, respectively). Compared to the control, *GIS2 *expression was increased 40-fold at the mRNA level and 12-fold at the protein level, which represents the highest fold-change in both mRNA and protein levels of all analyzed features, thus validating our experimental setup. There was minimal correlation between relative changes of mRNA and protein levels (Figure 6a; Pearson *r *< 0.1), possibly due to the observed mild effects on global mRNA and protein levels upon *GIS2 *overexpression. About 5% of all analyzed mRNAs and proteins changed relative expression by at least 1.5-fold with *P *< 0.05 (Additional file 9). Thereby, Gis2p targets are significantly overrepresented among the up-regulated transcripts and proteins (54 out of 205 up-regulated transcripts (26%, *P *= 10^-5^); and 11 out of 28 up-regulated proteins (40%, *P *= 0.025)). Some common functional attributes could also be assigned to the differentially expressed transcripts/proteins (see Additional files 10 and 11 for GO analysis of up- and down-regulated transcripts). Noteworthy, both myosin II binding proteins (Mlc1, Mlc2) in yeast were among the 28 up-regulated proteins (*P *< 0.0006).

To globally delineate the impact of Gis2p expression on the fate of mRNA targets and proteins levels, we further analyzed relative expression changes of Gis2p targets (FDR <5% in Gis2p affinity isolations) in the mRNA and protein expression datasets. RNA expression data were available for 973 (98%) of the 995 defined Gis2p target transcripts, and qMS data for 265 (27%) of the encoded proteins. Apparently, there is no 'simple' correlation between *GIS2 *expression and the fate of its targets, as the relative expression of the 973 Gis2 targets (FDR <5%) was neither commonly increased nor decreased in *GIS2 *overexpressing/mutant cells compared to all 'non-target' mRNAs (*P *~ 0.3) or proteins (*P *= 0.34). However, combinatorial binding of RBPs may greatly impinge on simple linear correlations between expression levels of RBPs and its targets [9]. Since RPBs often bind to functionally related target sets, we reasoned that such combinatorial control could lead to the partition of targets for an RBP into functionally related subsets with different outcomes for their mRNA fates (see later discussion). To address this, we analyzed expression changes of Gis2p targets within particular GO clusters (Figure 6b). Indeed, we found different and significant regulatory responses between functionally related subsets of mRNA targets on a global scale.

In a first instance, we found that mRNA levels for 104 Gis2p targets coding for rRNA processing factors (GO:0006364) were significantly increased in *gis2Δ *mutants compared to all genes (Figure 6b); these targets were also differentially expressed compared to the remaining 145 non-Gis2p targets annotated to this GO term (*P *= 0.0002, Mann-Whitney U test). Likewise, data were obtained for genes assigned to the nucleolus (GO:0005730), which is the cellular compartment were rRNA processing occurs; the 116 Gis2p targets were slightly up-regulated in *gis2Δ *mutants, and their expression differed significantly from 150 nucleolar non-Gis2p targets (*P *< 0.001, Mann-Whitney U test). Opposite to *gis2Δ *mutants, mRNA and protein levels of Gis2p targets coding for rRNA processing and nucleolar proteins were significantly decreased in *GIS2 *overexpressing cells (Figure 6b). In these cases however, the expression levels of non-Gis2p targets assigned to the respective GO terms were not significantly different from those of Gis2p targets (*P *= 0.07), although their average expression was slightly less diminished (delta log_2 _ratio = 0.05). Nevertheless, these results indicate that Gis2p selectively represses the expression of this group of messages, which could connect to the observed phenotypes regarding cell-size and growth.

In another instance, we found that the relative mRNA expression levels for proteins with GTPase activity (GO:0003924) were significantly lowered in *GIS2 *overexpressing cells, whereas levels of mRNAs coding for proteins involved in the establishment and maintenance of chromatin architecture (GO:0006325) were significantly increased upon *GIS2 *overexpression, exemplifying differential effects on different functional subsets of targets. Notably, in both of these cases, Gis2p targets were differentially expressed compared to non-Gis2p target genes within respective GO groups (GTPase activity, *P *= 0.03; chromatin architecture, *P *< 0.001; Mann-Whitney U test), further indicating specific effects on Gis2p targets within functionally related groups of messages. On the other hand, mRNA expression levels of these groups of messages were not significantly altered in *gis2Δ *cells. The regulatory effect on these messages may therefore reflect a short-term response upon *GIS2 *activation, which cannot be seen in *gis2 *mutant cells that have been grown for many generations, allowing adaptation to certain regulatory programs induced by the loss-of-function.

In a third instance, we recognized that levels of the myosin light chain proteins Mlc1, Mlc2, and type I myosin Myo3, which are all assigned to the GO term 'motor activity' (GO:0003774), were significantly increased upon *GIS2 *overexpression (mean log_2 _ratio = 0.9; *P *= 7 × 10^-4^), but the respective mRNA levels remained unchanged. Importantly, the protein levels of four non-Gis2p targets assigned to this GO term were not altered (mean log_2 _ratio = -0.06) and were differently expressed compared to Gis2p targets (*P *= 0.0032, Student's *t*-test). This indicates that Gis2p may specifically regulate translation of this related group of targets. In conclusion, these results show that Gis2p differentially affects the expression of functionally related subsets of targets at either the mRNA or protein level, suggesting strong coordinative functions of Gis2p for gene expression.

## Discussion

Based on our experimentally defined list of Gis2p mRNA targets and a series of RNA pull-down experiments, we were able to map the RNA-binding selectivity for Gis2p and human ZNF9. Rather unexpectedly, we found that both proteins have quasi-identical RNA-binding specificities, best interacting with RNAs that bear six to eight GWW repeats (W = A/U; Figure 3). Moreover, we provide data showing that Gis2p also binds to G-rich ssDNA, as previously reported for human ZNF9 [24]. We believe that these interactions are most likely established by all or a subset of the seven highly conserved CCHC-type ZnFs, where each of the ZnFs interacts with one GWW tri-nucleotide in RNA (Additional file 1). This scenario appears plausible considering previous detailed analysis of the RNA- and DNA-binding properties of ZnF proteins. Analogous to GWW repeats, certain classes of DNA-binding ZnF proteins contact the three adjacent nucleotides of GNN triplets [48]. Moreover, the closely related two CCHC knuckles of the nucleocapsid protein of HIV-1 specifically recognize an exposed guanosine base located in a single-stranded G-rich loop of the RNA [49]; and binding of ZnFs to AU-rich sequences in RNA has been demonstrated for human Tis11d, which bears two CCCH class ZnF domains [50]. On the other hand, the ZnF-dependent interaction with RNA/DNA by Gis2p/ZNF9 is somewhat in contradiction to a previous report that suggested strong involvement of the RGG box in ZNF9 for RNA binding [51]. Since the RGG box is exclusively present in human and vertebrates but absent in yeast and invertebrates (*Drosophila melanogaster *(CG3800), *Caenorhabditis elegans *(K08D12.3)), we speculate that the RGG box could direct interactions with other RNA elements, possibly to G-rich RNA elements located near TOP sequences in the 5'-UTR of messages encoding translation factors (note that TOP sequences are not present in yeast) [25-27]. Noteworthy, RNA binding through the RGG-box to G-rich elements has been found to be relatively weak (Kd about 10 μM) [24], and thus may require stabilization by other proteins *in vivo *as previously reported [26]. Further mutational analysis of protein motifs will be required to resolve the contribution of individual domains to RNA binding.

In accordance with the post-transcriptional operon model [10,52], we found common functional groups that were significantly enriched among the Gis2-associated mRNAs (Figures 1b and 4). Many of the yeast themes could directly relate to phenotypes, such as rRNA processing factors and components of the Ras pathway, which are known regulators of cell size. It appears that many of these functional groups are shared among predicted human targets. Such strong functional conservation among mRNA targets is distinct from previous findings with other evolutionarily conserved RBPs. In the case of the Pumilio/FBF (PUF) family proteins in yeast, flies and human, the PUF RNA-binding domain is well-conserved as is the sequence motif to which this domain binds; however, the functional attributes encoded by the bound messages dramatically diverged [8,53]. Possibly, this discordance may relate to the location of RBP-binding motifs: PUF proteins mainly bind to motifs located in 3'-UTRs, and we previously proposed that the acquisition or loss of RBP-binding motifs in UTRs provides a fluid evolutionary mechanism to modify post-transcriptional regulatory connections [8]. Contrarily, CDSs have been more conserved during evolution [54] and thus may provide a mechanism to preserve post-transcriptional regulatory networks. Nonetheless, the predicted ZNF9 targets additionally included mRNAs encoding proteins involved in muscle contraction, such as myosins, troponins, and ion channels, and for some of them we confirmed binding by ZNF9 with RNA pull-down assays (Figure 3d). Since ZNF9 is prominently expressed in heart and muscle in humans [28] these messages could be reached by ZNF9 for regulatory purposes. Whether and how ZNF9 impacts on these messages is not known, but if so, we hypothesize that altered ZNF9 activity could lead to altered 'coordination' of gene expression with potential consequences for muscle function and/or biogenesis. In support of these speculations are phenotypes seen in *ZNF9*^*+/- *^heterozygous mice that show some of the characteristic abnormalities of DM, including myotonia, ocular cataracts, cardiac arrhythmia, and both proximal and distal muscle wasting [34]. Likewise, the *ZNF9 *homolog in *C. elegans *(K08D12.3) is prominently expressed in muscle cells and knock-down mutants have defects in locomotion [55].

Our matched-sample proteome-transcriptome analysis showed that Gis2p does not linearly affect the expression of its mRNA targets, but rather adds differential effects to functionally related subsets (Figure 6). This adds to previous observations on the impact of RBPs on the fate of particular RNA targets, which can be controversial, possibly because other RBPs participate in the regulation of bound messages [3,45,56]. For example, Khd1p, a well-expressed yeast RBP that targets hundreds of messages, is required for translational repression of certain messages during transport to the bud-tip in dividing yeast cells; but it also positively affects expression of other messages through mRNA stabilization [45]. Indeed, comparing Gis2p RNA targets with the experimentally defined targets for more than 40 different yeast RBPs [9] reveals that RNA targets for 12 RBPs (Cbc2, Gbp2, Msl5, Nab2, Npl3, Nrd1, Nsr1, Pab1, Pin4, Puf5, Puf5, Vts1) significantly overlap with Gis2p targets, indicating great potential for combinatorial control (Additional file 12 depicts the overlap of Gis2p targets with RNA targets for 36 RBPs). Most prominent, 68% of the RNA targets of Gbp2 - a shuttling RBP involved in mRNA export and associated with ribosomes [57,58] - were among our experimentally defined Gis2p targets (*P *< 10^-132^, hypergeometric distribution). This subset comprises 259 annotated gene transcripts; a substantial fraction of them (46 genes, 17.8%) code for proteins acting in rRNA processing (*P *< 3 × 10^-15^). Likewise, 36% of Nsr1p RNA targets were associated with Gis2p (384 of 1,049 Nsr1 targets, *P *< 10^-64^). Nsr1 is a nucleolar protein required for pre-rRNA processing and ribosome biogenesis [59]. A significant fraction of the messages bound by Nsr1 and Gis2 proteins code for structural components of the ribosomes (35 genes, *P *< 2 × 10^-7^) or have pyrophosphatase activity (40 genes, *P *< 5 × 10^-5^), which includes 13 GTPases (*P *< 0.0003) covering most of the Gis2p GTPase target messages (65%, 13 of 20 GTPases with Nsr1 data). Whether and how such combinatorial arrangement may impinge on the fate of particular mRNAs is not known at this point, but it becomes apparent that strong combinatorial control on Gis2p targets is very likely and - as we speculate - may represent a source for differential outcome of their fates.

On the other hand, differential outcomes of mRNA fates may also be directed by temporally or spatially restricted direct interactions of Gis2p with other factors that participate in RNA regulation. In this regard, data from large-scale yeast interactome analysis suggests that Gis2 interacts with at least 28 RBPs that play a role in translation, RNA decay or rRNA processing (in total, 48 physical or genetic interactions are reported in SGD as of October 2010). For instance, Gis2p physically interacts with the translation initiation factor 2A (Ygr054p) and the cytoplasmic ATP-dependent RNA helicases Dbp1, Dbp2, and Dhh1, as well as with Lsm1 and Lsm6, which are found in association with the Pat1 decapping enzyme and Xrn1 exoribonuclease involved in mRNA degradation. There are also diverse reports on physical and genetic interactions of Gis2 with rRNA processing factors (Nop1, Pop1, Pop7, Pwp1, Prp7, Gar1 and others), which coincidences with our observed preference of Gis2p to target related transcripts coding for proteins acting in this process. Considering this strong bias for interaction partners acting in RNA-related processes, it appears plausible that Gis2p could connect RNA regulons to different layers of post-transcriptional gene regulation (for example, translation/decay). The observed regulatory impact on specific RNAs may be relatively weak with respect to the amplitude of regulation, but it may still become biologically significant when considering all messages in a RNA regulon. Such coordinate function for gene expression may be important for fine-tuning the expression of functionally related groups, complexes, or pathways. It could also provide a means to reduce arbitrary 'fluctuations' or 'noise' within gene clusters, tightly balancing transcript and protein levels. In this regard, the compensative changes in gene expression may be preferentially noticed upon short-term changes of RBP expression, as some regulatory patterns could be more readily seen after short-term induction of Gis2 expression than after prolonged depletion. Lastly, we wish to note that besides acting as a post-transcriptional 'coordinator', we cannot exclude that Gis2p may also act as a transcriptional regulator under certain circumstances as we have shown potential binding to ssDNA. However, to our knowledge no direct physical interactions of Gis2p with DNA-binding factors have been reported in the SGD. Since Gis2p is preferentially localized to the cytoplasm, such function may involve re-localization to the nucleus, which could be directed by post-translational modification of the protein. Further investigations are required to delineate the molecular details of Gis2p function in gene expression.

## Conclusions

In this study, we provide an integrated analysis defining potentially conserved RNA regulons for homologous ZnF proteins in yeast and human. These predictions were defined by repetitive sequence elements located in CDSs of messages, which is different to RBPs that bind to sequence elements located in UTRs. In addition, our analysis provokes speculation about an alternative role of human ZNF9 in the regulation of muscle-specific genes, which may be linked to DM-related phenotypes seen in *ZNF9 *mouse models. Finally, we established global effects of Gis2 expression on mRNA targets, showing that Gis2p does not linearly affect their expression but rather differentially effects functional subsets of its targets, first exemplifying distinct coordinative roles for RPBs on functional target groups.

This analysis highlights the potential of combining RNA-binding protein immunopurification-microarray (RIP-Chip), transcriptome and proteome analysis in order to generate an integrated view of gene networks that are coordinated by RBPs. In the future, this approach may be used to decipher the structure and plasticity of RNA regulons met by RBPs in a wide range of species, following reactions of internal and external stimuli that will reveal conserved and divergent response pathways.

## Materials and methods

### Yeast strains, plasmids, and media

TAP-tagged Gis2 (YNL255) [18], the isogenic wild-type strain BY4741 (MATa *his3Δ1 leu2Δ0 met15Δ0 ura3Δ0*) and a *GIS2 *deletion strain (*gis2Δ*) were obtained from Open Biosystems (Huntsville, AL, USA). Plasmids pBG1805-Gis2 and pBG1805-Slf1 for expression of tagged-proteins under the control of a galactose inducible promoter [60] were from Open Biosystems; pBG1805-ZNF9 was constructed by Gateway subcloning (Invitrogen, Paisley, UK) of the CDS of *ZNF9 *from pDONR-221-ZNF9 into pBG1805. Plasmids for expression of (His)_6_-tagged fusion proteins in *E. coli *were generated as follows. The CDS of *GIS2 *was subcloned from plasmid pBG1805-Gis2 into pDONR-221 by Gateway cloning (Invitrogen). The CDS of human *ZNF9 *was amplified by PCR from HeLa cDNA with complementary primers ZNF9-Fw and ZNF9-Rev, which contain Gateway compatible ends (a list of primer sequences and PCR products is given in Additional file 13). PCR products were cloned into pDONR-221 and sequenced, which revealed the ZNF9 isoform 2 (NP_001120665.1). The CDSs of *GIS2 *and *ZNF9 *were excised from the pDONR-221 and subcloned into the *E. coli *expression vector pDEST-17 (Invitrogen) by Gateway cloning, generating plasmids pDEST-17-Gis2 and pDEST-17-ZNF9, respectively. The media to cultivate yeast cells included rich medium (YPD) and synthetic complete (SC) medium [61]. SC media lacking amino acids or other nutrients (SC -Ura corresponds to SC lacking uracil) were used to select transformants. SR and SG media were identical to SC except that they contained raffinose and galactose, respectively, instead of 2% glucose.

### RNA immunoaffinity isolations

Affinity purification of Gis2-TAP together with the associated RNAs was carried out essentially as described [7]. One-liter cultures of a strain expressing Gis2-TAP or the wild-type strain BY4741 (mock control) were grown to an optical density at 600 nm of 0.6 to 0.9 in YPD at 30°C. Cells were harvested by centrifugation, washed twice with 25 ml of ice cold buffer A (20 mM Tris-HCl (pH 8.0), 140 mM KCl, 1.8 mM MgCl_2_, 0.1% Nonidet P-40, 0.02 mg/ml heparin), frozen in liquid nitrogen, and stored at -80°C. The cells were thawed at 4°C and resuspended in 5 ml of buffer B (buffer A plus 0.5 mM DTT, 1 mM phenylmethanesulphonylfluoride (PMSF), 1 μg/ml leupeptin, 1 μg/ml pepstatin, 20 U/ml DNase I (Roche Diagnostics GmbH, Mannheim, Germany) 50 U/ml RNasin (Promega. Madison, WI, USA) 0.2 mg/ml heparin), and mechanically broken with glass beads with a Tissue Lyser (Qiagen, Hilden, Germany) for 12 minutes at 300 Hz and 4°C. The lysate was centrifuged sequentially for 5 minutes at 2,000 g, 12,000 g, and 16,000 g at 4°C to remove cell debris. The volume of the extract was adjusted to 5 ml with buffer B and a 100 μl aliquot was removed for reference RNA isolation. The remaining 4.9 ml extract was incubated with 500 μl 50% (v/v) IgG-agarose beads (Sigma-Aldrich, St. Louis, MO, USA) in buffer A for 2 h at 4°C under gentle rotation of tubes. The beads were washed once with 5 ml buffer B for 15 minutes, and three times with 12 ml buffer C (20 mM Tris-HCl (pH 8.0), 140 mM KCl, 1.8 mM MgCl_2_, 0.5 mM DTT, 0.01% NP-40, 15 U/ml SUPERase•In (Applied Biosystems/Ambion, Austin, TX, USA; catalogue number AM2694), 1 μg/ml pepstatin, 1 μg/ml leupeptin, 1 mM PMSF) for 15 minutes with gentle agitation. The beads were transferred into 1.2-ml micro-spin columns (BioRad, Hercules, CA, USA; catalogue number 732-6204) and pelleted by centrifugation for 2 minutes at 6,000g in a table-top centrifuge. The supernatant was removed and the beads were resuspended in 1.5× bed volumes (375 μl) of buffer C. TAP-tagged Gis2 was released from the beads by incubation with 80 U of acTEV protease (Invitrogen, catalogue number 12575023) for 2 h at 16°C. RNA from the extract (input) and from the affinity isolates was purified with the RNeasy Mini/Micro Kit (Qiagen). Three independent affinity purifications were performed with the Gis2-TAP strain and five mock control isolations with the untagged wild-type strain.

### Growth of *gis2Δ *mutants and *GIS2 *overexpressing cells

Fifty milliliters of *gis2Δ *cells and the respective wild-type strain BY4741 were grown in YPD at 30°C to an OD_600 _of 0.5 to 0.6. Cells were harvested by centrifugation and washed twice with 800 μl of bi-distilled water, and RNA was isolated by hot phenol extraction. For *GIS2 *overexpression, 100 ml of BY4741 cells bearing plasmid pBG1805-Gis2 or the empty plasmid pBG1805 (control) were grown in SR -Ura medium at 30°C to an OD_600 _of 0.5 to 0.7 and induced with 2% galactose for 1.5 h before harvesting. Total RNA was isolated from 100 μl (1/8) of cells, and the residual cells were resuspended in 200 μl of urea buffer (8 M urea, 0.1 M ammonium bicarbonate) and lysed with glass beads in a Tissue Lyser (Qiagen). The lysate was cleared by centrifugation at 3,000g for 2 minutes at 4°C. To maximize the recovery of proteins, the pellets containing glass beads were washed once with 60 μl of urea buffer. The combined lysate was centrifuged at 12,000g and 16,000g for 5 minutes each at 4°C. Protein concentrations were determined with the Bicinchoninic Acid (BCA) Kit (Thermo Fisher Scientific, Rockford, IL, USA) with BSA (Sigma-Aldrich) as a reference standard. The extracts were frozen in liquid nitrogen and stored at -80°C. Two biological replicates and two technical replicates were analyzed with microarrays, and three biological replicates were analyzed by qMS.

### DNA microarray analysis

Microarray data are available at the Stanford Microarray Database (SMD) [62] or at the Gene Expression Omnibus with accession number [GEO:GSE24005]. Yeast oligo arrays that contained probes for all annotated yeast ORFs, non-coding RNAs, and some intergenic regions were processed and hybridized with fluorescently labeled cDNAs as described previously [63]. For affinity purifications, 5 μg of total RNA from the extract (input) and up to 50% (approximately 500 ng) of the affinity purified RNA were reverse transcribed in the presence of 5-(3-aminoallyl)-dUTP and natural dNTPs with a mixture of N9 and dT20V primers, and cDNAs were covalently linked to Cy3 and Cy5 NHS-monoesters (GE Healthcare, Little Chalfont, UK), respectively, and competitively hybridized on microarrays at 42°C for 14 h in formamide-based hybridization buffer. Likewise, 7.5 μg of total RNA was labeled for gene expression profiling of *GIS2 *overexpressing cells or *gis2Δ *mutants compared to control cells. Microarrays were scanned with an Axon Scanner 4200 (Molecular Devices, Sunnyvale, CA, USA) and analyzed with GenePix Pro 5.1 (Molecular Devices). Arrays were deposited and computer normalized at the SMD [62]. To identify transcripts that were specifically enriched with Gis2p, the log_2 _median ratios from three independent Gis2p affinity isolations and five mock control isolations were retrieved from SMD and exported into Microsoft Excel after filtering for signal over background >1.8 in the channel measuring total RNA derived from the extract. Two-class SAM (version 3.02) [35] was performed using *t*-test statistics on median centered arrays [8,53], and features with a FDR <5% were considered to be significantly associated with Gis2p (Additional file 2). To analyze mRNA expression profiles of *GIS2 *overexpression and *gis2Δ *mutants, the log_2 _median ratios from three biological replicates were filtered for regression correlation <0.5 and signal over background >1.5 in both channels. In analogy to the proteome profiling, SGD annotated features (January 2009) that changed expression at least 1.5-fold (average log_2 _ratio 0.585) with a *P*-value <0.05 (one-sample *t*-test with Acuity 4.0 (Molecular Devices)) were considered to be significantly changed.

### Label-free quantitative mass spectrometry

We applied a recently established technique that is based on the label-free quantification of peptide patterns acquired by high mass accuracy mass spectrometry [64]. This method performs with similar reproducibility and sensitivity as the Isotope-Coded Affinity Tags (ICAT) methodology, but does not depend on incorporation of isotope-labeled amino acids. qMS was essentially performed as described previously [64]: 200 μl of protein extract (2.5 mg/ml) was reduced with 5 mM Tris [2-carboxyethyl] phosphine (Thermo Fisher Scientific/Pierce, catalogue number 20490) for 30 minutes at 37°C and alkylated with 10 mM iodoacetamide (Sigma-Aldrich, catalogue number A3221) for 30 minutes at room temperature. The protein sample was diluted 1:4 with 100 mM ammonium bicarbonate (pH 7.8) and digested overnight with 20 μg of trypsin (Promega) at 37°C. The protein digest was acidified with formic acid to pH 2.5, and the sample was loaded onto a reversed phase C-18 Sep-Pak column (Waters, Milford, MA, USA); material, 100 mg; capacity, 1 mg) equilibrated in washing buffer (5% acetonitrile, 0.1% formic acid in ddH_2_O). The flow-through was re-loaded to minimize loss of peptides. The column was washed with 2 ml of washing buffer and the peptides were eluted with 700 μl of 80% acetonitrile containing 0.1% formic acid in ddH_2_O. The samples were dried in a SpeedVac (Thermo Fisher Scientific and re-solubilized in 100 μl of 0.1% formic acid in ddH_2_O for mass spectrometric analysis. The liquid chromatography tandem mass spectrometry (LC-MS/MS) analysis was performed on a hybrid linear ion trap Fourier-transform ion cyclotron resonance mass spectrometer (Thermo Electron, San Jose, CA, USA) that was interfaced to a nanoelectrospray ion source (both from Thermo Electron) coupled online to a Tempo 1D-plus nanoLC (Applied Biosystems/MDS SCIEX, Foster City, CA, USA). Peptides were separated on a RPLC column (75 μm × 15 cm) packed in-house with C18 resin (Magic C18 AQ 3 μm; Michrom BioResources, Auburn, CA, USA) using a linear gradient from 98% solvent A (98% water, 2% acetonitrile, 0.15% formic acid) and 2% solvent B (98% acetonitrile, 2% water, 0.15% formic acid) to 30% solvent B over 90 minutes at a flow rate of 0.3 ml per minute. MS/MS spectra were searched using SORCERER-SEQUEST v3.0.3, running on SageN Sorcerer, and using SGD (version of June 6 2008, which contains 6,760 protein entries). Trypsin was specified for cleavage, allowing for two missed cleavages and one non-tryptic terminus. Mass tolerance was set to 25 ppm for monoisotopic precursor ions, and to 0.5 Da for fragment ions. Stable modification for Cys of +57.0214 Da was used. The identified peptides were processed and analyzed through the mass spectrometry Trans-Proteomic Pipeline 3.5 (TPP) [65]. The LC-MS/MS data were quantified by integrating the peak area of the detected peptides using the software *SuperHirn *[66] as previously described [64].

### Expression of His-tagged fusion proteins in *E. coli*

BL-21 (DE3) cells bearing plasmids pDEST-17-Gis2 or pDEST-17-ZNF9 were grown to mid-log phase in LB broth supplemented with 100 μg/ml ampicillin (Sigma) at 37°C. Expression of the His-tagged fusion proteins was induced by the addition of 1 mM isopropyl-β-D-thiogalactopyranosid (IPTG, Sigma) for 4 h at 30°C. Cells were washed twice with ice-cold 50 mM phosphate buffer (pH 8.0), resuspended in 10 ml of lysis buffer per 2.5 g of cell pellet (50 mM phosphate-buffer pH 8.0, 50 mM NaCl, 10% glycerol, 25 mM imidazole), and lysed on ice with a sonicator (Sonoplus from Bandelin electric, Berlin, Germany; 2 × 10 minutes, 60% power). The cell lysate was cleared by centrifugation at 15,000g for 30 minutes at 4°C. The extract was loaded onto a nitrilotriacetic acid-Ni^2+ ^sepharose column and His-tagged proteins were eluted with lysis buffer supplemented with 200 mM imidazole pH 8 as specified by the manufacturer (GE Healthcare).

### Expression of tagged ZNF9 in yeast

BY4741 cells transfected with pBG1805-ZNF9 were grown in SR -Ura medium to an OD_600 _of 0.5 to 0.7. ZNF9 expression was induced by the addition of 2% galactose to the media and cells were further grown for 2 h. Cells were collected and extracts were prepared in IP buffer (50 mM phosphate buffer pH 8.0, 75 mM NaCl, 5% glycerol, 2 mM MgCl_2, _1 mM EDTA pH 8.0, 0.1% Triton X-100, 1 mM DTT, 1 mg/ml BSA, 0.1 mg/ml heparin, 0.1 mg/ml *E. coli *tRNA (Roche), 1 mM PMSF, 1 μg/ml leupeptin (Sigma), 0.8 μg/ml pepstatin (Sigma), 40 U/ml RNasin (Promega)) with glass beads as described above.

### Synthesis of biotinylated RNAs and RNA pull-down experiments

DNA templates for biotin-RNA synthesis were either prepared by PCR from *S. cerevisiae *or human DNA with oligonucleotides bearing a T7 RNA polymerase promotor sequence at their 5' ends, or by direct annealing of complementary oligonucleotide pairs containing a 5' T7 RNA polymerase promotor (a list of oligonucleotide primers is given in Additional file 13). For the annealing of oligos, a mixture of 1 μM of forward and reverse complementary oligonucleotides was incubated for 1 minute at 95°C in 20 μl of water and cooled down slowly to room temperature. Biotin-labeled RNAs were prepared with the Biotin RNA Labeling Mix (Roche) and T7 RNA polymerase (Promega). Biotinylated RNAs were treated with RNase-free DNase I and purified with the PureLink Micro-to-Midi Kit (Invitrogen). RNAs smaller than 60 nucleotides were purified with the mirVana PARIS Kit (Ambion, catalogue number AM1556). RNA was quantified by UV spectrometry with a Nanodrop device (Witec AG, Littau, Switzerland), and the integrity of the RNA was controlled by agarose gel electrophoresis.

RNA pull-downs were performed in IP buffer essentially as described [8]. Biotinylated RNAs (9 pmol) were combined with 50 μl of yeast extract (OD_280 _approximately 10) prepared from cells expressing either Gis2-TAP or tagged ZNF9, or with purified His-tagged proteins expressed in *E. coli *(50 ng/μl). The integrity of transcripts derived from PCR templates was controlled with RT-PCR after incubation with extracts; there was no indication of substantial degradation (data not shown). Streptavidin-captured RNA-protein complexes were resolved by SDS-PAGE and subjected to immunoblot analysis using PAP reagent (1:5,000; Sigma) to detect protein A-tagged proteins. His-tagged proteins were detected with anti-His antibodies (1:1,000; Qiagen) and horseradish peroxidase-coupled secondary antibodies (1:5,000). Blots were developed with the ECL Plus Western Blotting Detection System (GE Healthcare/Amersham) and bands were visualized on a film. Representative experiments from multiple replicates are shown in Figures 2 and 3.

### Phenotype studies

*Gis2Δ *cells or BY4741 wild-type cells were transformed with either pBG1805 (empty vector control), pBG1805-Gis2, pBG1805-ZNF9, or pBG1805-Slf1 and grown in SR -Ura medium at 30°C to an OD_600 _of 0.5 under constant agitation. For plate assays, cultures were serially diluted (1:5) and spotted onto SC or SG plates. To measure cell size, cells transformed with the respective plasmids were grown in SG -Ura medium at 30°C to an OD_600 _of 1.2 and examined on a microscope slide with an Axioskop 2 Mot Plus microscope (Carl Zeiss AG). Images were captured at 40× magnification with an AxioCam MRm digital camera and analyzed with Axio Vision 4.4 software (Zeiss). Adobe Photoshop CS4 (Adobe Systems, San Jose, CA, USA) was used for quantification of cell size using the quick selection tool (measurement scale: 1 pixel = 0.155 μm). A two-tailed Student's *t*-test implemented in GraphPad Prism version 5.0 for Windows was used for statistics (GraphPad Software, San Diego, CA, USA).

### Bioinformatic tools

Yeast ORF CDSs were downloaded from SGD, and UTR sequences for 5,880 yeast transcripts were taken from [43]. To search for motifs within nucleotide sequences, the sense strand was searched with MEME under the default settings [67]. The number and location of consensus sequences among yeast ORF CDSs was obtained by searching with PatMatch in SGD [42]. The occurrence of GWW motifs within yeast UTRs was analyzed with an Excel implemented Pearl Script. Human CDSs were downloaded with BioMart (ENSEMBL v48). RNA structures were predicted with Mfold [68,69]. Significantly shared GO terms among sets of genes were identified with the GO term enrichment tool at the AMIGO GO database (December 2008/October 2010 versions) [38,70] considering all SGD and UniprotKB (The Universal Protein Resource Consortium, UniProt) annotated gene sets as background database filters for yeast and human genes, respectively. For the discovery of up- and down-regulated GO gene sets, we used GOrilla [41,71]. FunSpec analysis of Gis2p targets (FDR <5%) was performed via the web-based program [39,72].

## Abbreviations

BSA: bovine serum albumin; CDS: coding sequence; DM: myotonic dystrophy; DTT: dithiothreitol; FDR: false discovery rate; GO: Gene Ontology; Gorilla: Gene Ontology enrichment analysis and visualization; LC-MS/MS: liquid chromatography tandem mass spectrometry; MEME: Multiple Expectation Maximization for Motif Elicitation; ORF: open reading frame; PMSF: phenylmethanesulphonylfluoride; qMS: quantitative mass spectrometry; RBP: RNA-binding protein; SAM: Significance Analysis of Microarrays; SGD: *Saccharomyces *Genome Database; SMD: Stanford Microarray Database; snoRNA: small nucleolar RNA; SC: synthetic complete; ssDNA: single-stranded DNA; TAP: tandem-affinity purification; TOP: terminal oligopyrimidine; UTR: untranslated region; ZnF: zinc finger.

## Authors' contributions

TS designed and performed experiments, analyzed data and wrote the manuscript. CF performed RNA pull-downs, cloned and expressed *ZNF9*, and initiated the transcriptome-proteome analysis. RS performed the qMS analysis. RA gave advice and supported the qMS analysis. APG designed experiments, analyzed data and wrote the manuscript.

## Supplementary Material

Additional file 1 **Domain structure and amino acid sequence alignment of Gis2p homologs**. **(a) **Domain structure. CCHC-type ZnFs are shown in blue, the RGG-box in yellow. **(b) **Multiple amino acid sequence alignment of ZNF9 homologs in vertebrates and yeast Gis2p. The seven CCHC zinc-knuckle motifs are boxed in black, the RGG-motif is boxed in black with a transparent white background. A color code representing the physiochemical properties of amino acids is shown above the alignment. Entrez Protein Database accession numbers: [AAI02299] (*Bos taurus*), [AAD33937] (*Bufo arenarum*), [AAO73520] (*Danio rerio*), [AAB62243] (*Gallus gallus*), [AAA61975] (*Homo sapiens*), [AAB60490] (*Mus musculus*), [BAA08212] (*Rattus norvegicus*), [CAA69031] (*Xenopus laevis*), [AAI22021] (*Xenopus tropicalis*), and [AAS56328] (*Saccharomyces cerevisiae*). Click here for file

Additional file 2 **List of Gis2p RNA targets**. Columns indicate the following (from left to right): ORF; gene name; GO annotations; SAM-score; FDR; (GWW)_4 _repeat in the CDS; average log_2 _ratio and *P*-value of relative changes of mRNA levels upon *GIS2 *overexpression; average log_2 _ratio and *P*-value of relative changes of protein levels upon *GIS2 *overexpression. Click here for file

Additional file 3 **Raw data and SAM analysis of Gis2p affinity isolates**. **(a) **Log_2 _ratios of enrichment in three independent Gis2-TAP affinity isolations and five mock control isolations. **(b) **SAM analysis with *q*-value representing FDRs. Click here for file

Additional file 4 **Significantly shared GO terms among Gis2p mRNA targets**. GO enrichment analysis on the 995 messages associated with Gis2p with FDR <5% with AMIGO, or with GOrilla on SAM-ranked data. If multiple related GO terms were retrieved for the same set of genes, only the most significant terms are shown. GOrilla enrichment = (b/n)/(B/N); N, total number of genes in the reference database; B, number of genes in the reference database associated with a specific GO term; n, number of genes in the target dataset; b, number of genes in the target set associated with a specific GO term. Click here for file

Additional file 5 **Distribution of ORFs with (GAN)**_**7 **_**repeats in Gis2p affinity isolates**. The number of (GAN)_7 _motifs is plotted on the *y*-axis and ORFs are ranked on the *x*-axis based on decreasing SAM scores for Gis2p association. Range of genes with FDR <5% are marked with a bar. The numbers of (GAN)_7 _repeats as well as the number of genes with at least one (GAN)_7 _repeat in the ORF are indicated for Gis2p targets (red) and non-targets (green). Click here for file

Additional file 6 ***S. cerevisiae *CDSs with GWW repeats of various lengths**. Click here for file

Additional file 7 **Human CDSs with GWW repeats of various lengths representing predicted ZNF9 targets**. Click here for file

Additional file 8 **Microarray data representing relative changes (log**_**2 **_**ratio) of RNA levels of *gis2Δ *versus wild-type cells (BY4741)**. Experimentally defined Gis2p targets are marked in a separate column. A list of significantly up-regulated and down-regulated genes (1.5-fold, *P *< 0.05) and a GO enrichment analysis for these genes are given in separate worksheets. Click here for file

Additional file 9 **Venn diagram representing mRNAs/proteins that changed upon *GIS2 *overexpression and in *gis2Δ *mutants**. The number of all analyzed mRNAs/proteins is indicated in purple, and the number of experimentally defined Gis2p targets (FDR <5%) is shown in blue. The number of features for which relative expression was selectively increased is indicated within the red circle, features with decreased expression are within green circles (cutoff: mRNAs/proteins that changed at least 1.5-fold, *P *< 0.05). *P*-values relate to the significance of the overlap (Chi square test). Click here for file

Additional file 10 **Microarray data representing relative changes (log**_**2 **_**ratio) of RNA levels in *GIS2 *overexpressing versus control cells**. Data for two biological replicates (1 and 2) and two technical replicates (a and b) are shown in different columns. Experimentally defined Gis2p RNA targets are indicated in a separate column. A list of significantly up-regulated and down-regulated genes (1.5-fold, *P *< 0.05) and a GO enrichment analysis are given in separate worksheets. Click here for file

Additional file 11 **Raw qMS data comparing protein levels from cells overexpressing *GIS2 *with control cells**. Experimentally defined Gis2p targets are indicated in a separate column. A list of significantly up-regulated and down-regulated genes (1.5-fold, *P *< 0.05) and a GO enrichment analysis are given in separate worksheets. Click here for file

Additional file 12 **Overlap between RNA targets for Gis2p and 36 yeast RBPs**. Data for RNA targets for yeast RBPs with FDR <5% were retrieved from Hogan *et al*. [9]. Thirty-six RBPs, which had at least eight RNA targets, were further considered for this analysis. The number of targets shared with Gis2p and the number of all targets are indicated next to the name of the RBP. The histogram depicts the fraction of Gis2p targets among RBP targets. Significant overlaps are marked with asterisks (hypergeometric distribution with Bonferroni correction; ***P < 10^-10^, **P < 10^-5^, *P < 0.01). Click here for file

Additional file 13 **Oligonucleotide primer sequences**. Click here for file
