# Supplementary material for: Defining potentially conserved RNA regulons of homologous zinc-finger RNA-binding proteins
Source: Genome Biol. 2011 Jan 13;12(1):R3. doi: 10.1186/gb-2011-12-1-r3 (PMC3091301; doi:10.1186/gb-2011-12-1-r3)
Supplement: Additional file 1 — Domain structure and amino acid sequence alignment of Gis2p homologs. (a) Domain structure. CCHC-type ZnFs are shown in blue, the RGG-box in yellow. (b) Multiple amino acid sequence alignment of ZNF9 homologs in vertebrates and yeast Gis2p. The seven CCHC zinc-knuckle motifs are boxed in black, the RGG-motif is boxed in black with a transparent white background. A color code representing the physiochemical properties of amino acids is shown above the alignment. Entrez Protein Database accession numbers: [AAI02299] (Bos taurus), [AAD33937] (Bufo arenarum), [AAO73520] (Danio rerio), [AAB62243] (Gallus gallus), [AAA61975] (Homo sapiens), [AAB60490] (Mus musculus), [BAA08212] (Rattus norvegicus), [CAA69031] (Xenopus laevis), [AAI22021] (Xenopus tropicalis), and [AAS56328] (Saccharomyces cerevisiae). [file gb-2011-12-1-r3-S1.PDF]

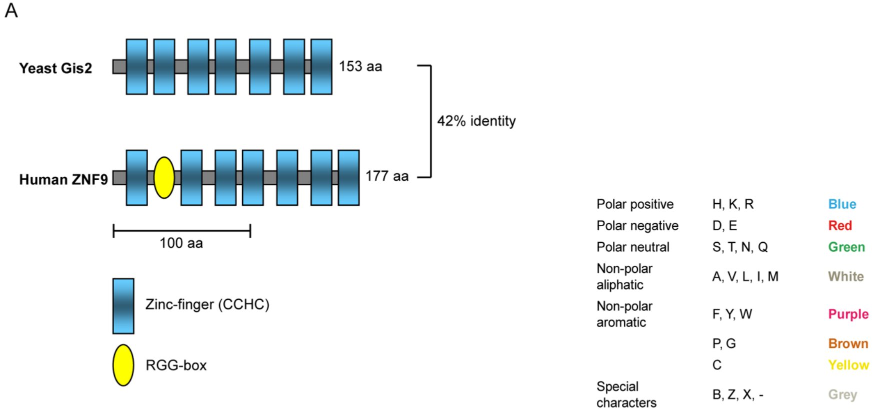
A
Yeast Gis2
153 aa
42% identity
Human ZNF9
177 aa
100 aa
Zinc-finger (CCHC)
RGG-box
Polar positive H, K, R Blue
Polar negative D, E Red
Polar neutral S, T, N, Q Green
Non-polar aliphatic A, V, L, I, M White
Non-polar aromatic F, Y, W Purple
P, G Brown
C Yellow
Special characters B, Z, X, - Grey

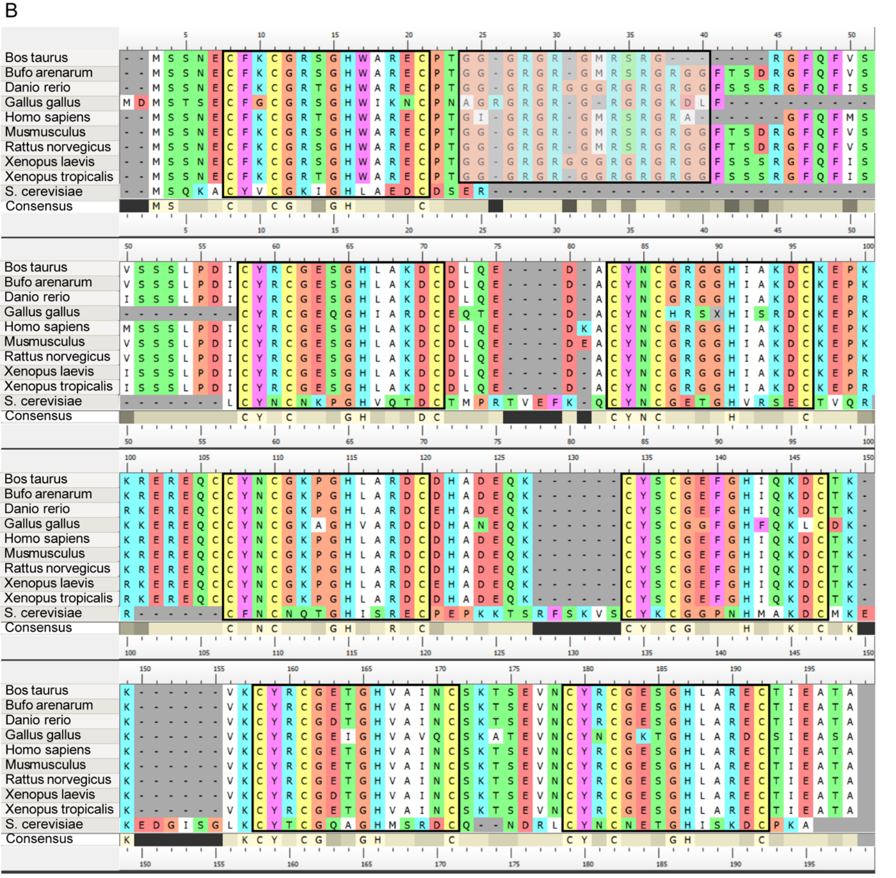
B
Bos taurus
Bufo arenarum
Danio rerio
Gallus gallus
Homo sapiens
Musmusculus
Rattus norvegicus
Xenopus laevis
Xenopus tropicalis
S. cerevisiae
Consensus
