# Supplementary figures and images for: Defining potentially conserved RNA regulons of homologous zinc-finger RNA-binding proteins
Source: Genome Biol. 2011 Jan 13;12(1):R3. doi: 10.1186/gb-2011-12-1-r3 (PMC3091301; doi:10.1186/gb-2011-12-1-r3)

*GIS2*  
overexpression

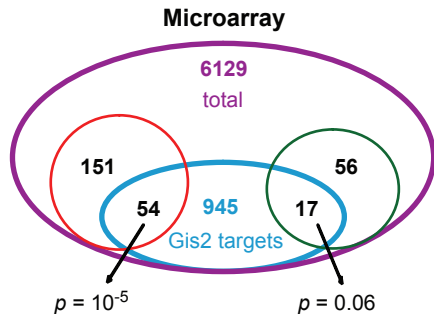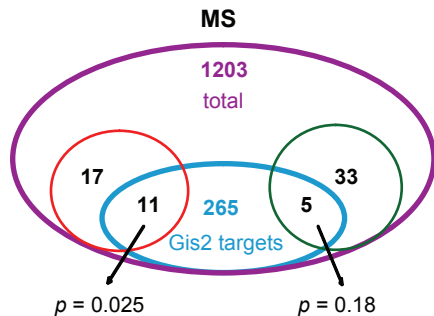

*gis2Δ*  
mutant

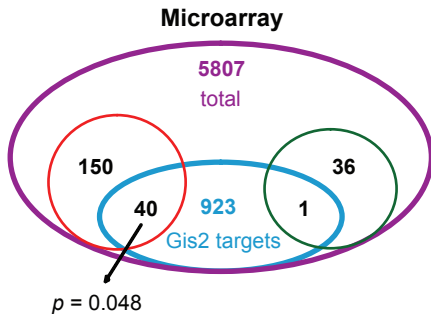

— up } 1.5 fold  
— down }  $p < 0.05$

Supplement: Additional file 9 — Venn diagram representing mRNAs/proteins that changed upon GIS2 overexpression and in gis2Δ mutants. The number of all analyzed mRNAs/proteins is indicated in purple, and the number of experimentally defined Gis2p targets (FDR <5%) is shown in blue. The number of features for which relative expression was selectively increased is indicated within the red circle, features with decreased expression are within green circles (cutoff: mRNAs/proteins that changed at least 1.5-fold, P < 0.05). P-values relate to the significance of the overlap (Chi square test). [file gb-2011-12-1-r3-S9.PDF]

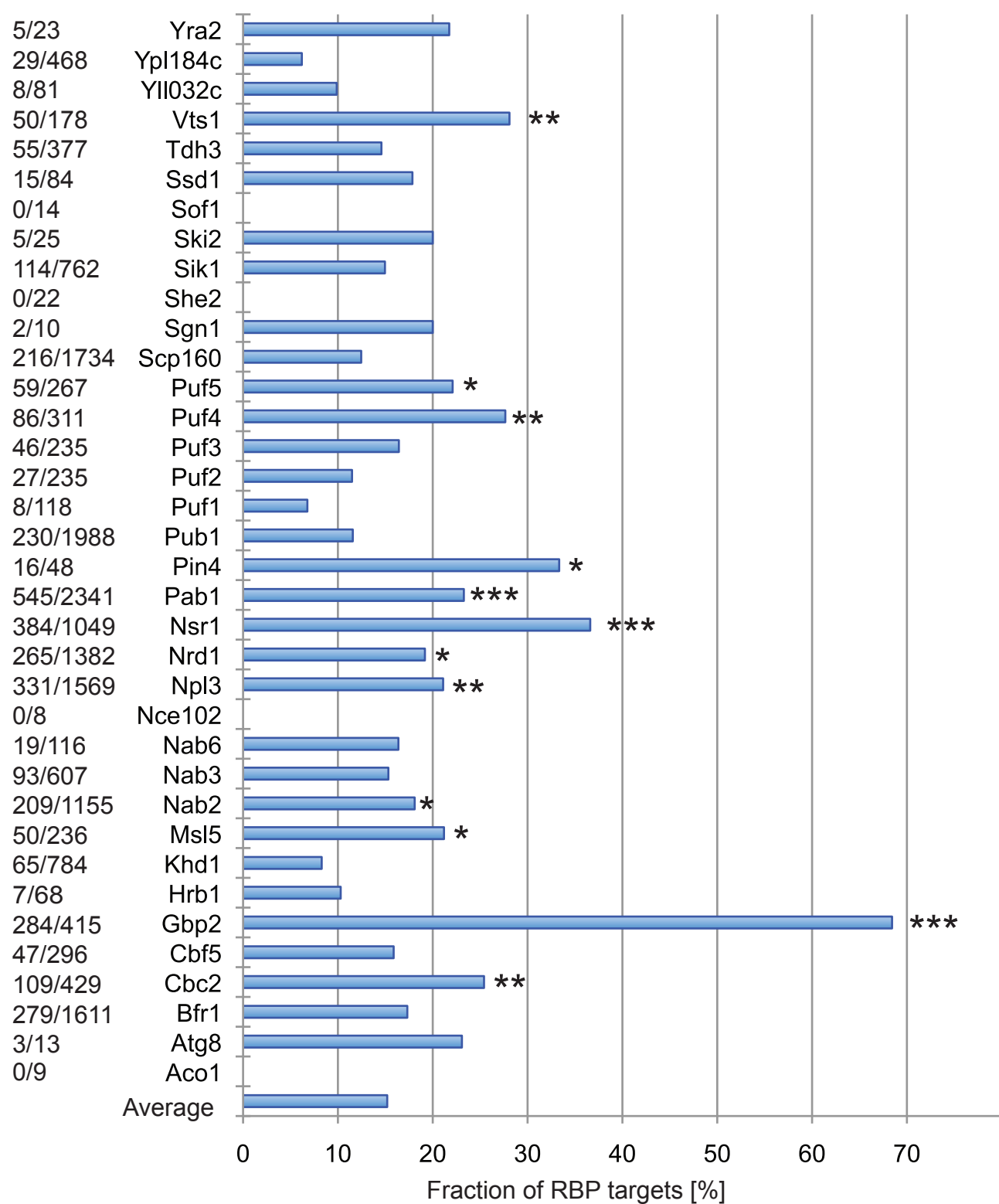

Supplement: Additional file 12 — Overlap between RNA targets for Gis2p and 36 yeast RBPs. Data for RNA targets for yeast RBPs with FDR <5% were retrieved from Hogan et al. [9]. Thirty-six RBPs, which had at least eight RNA targets, were further considered for this analysis. The number of targets shared with Gis2p and the number of all targets are indicated next to the name of the RBP. The histogram depicts the fraction of Gis2p targets among RBP targets. Significant overlaps are marked with asterisks (hypergeometric distribution with Bonferroni correction; ***P < 10-10, **P < 10-5, *P < 0.01). [file gb-2011-12-1-r3-S12.PDF]
