# Supplementary material for: Defining potentially conserved RNA regulons of homologous zinc-finger RNA-binding proteins
Source: Genome Biol. 2011 Jan 13;12(1):R3. doi: 10.1186/gb-2011-12-1-r3 (PMC3091301; doi:10.1186/gb-2011-12-1-r3)
Supplement: Additional file 13 — Oligonucleotide primer sequences. [file gb-2011-12-1-r3-S13.PDF]

Additional file 13.

Oligonucleotide primers and PCR reactions.

The following PCR primers were used to generate templates for *in vitro* transcription (T7 promoter is underlined). Yeast genomic DNA of BY4741 strain was used as template for PCR with Taq Polymerase (GoTaq®, Promega Cat.# M3175).

Primer Name	Sequence	PCR product length [nt]
T7-Erv25-3UTRFw	TAATACGACTCACTATAGGGAAGCAAAGGAAGAGGAC	542
Erv25-3UTRRev	TGCAACTAGAGCCCACAATTTGGAAACTGG	see Fw primer
T7-Erv25-5UTRFw	TAATACGACTCACTATAGGGCCATGACTACACTTTACC	668
Erv25-5UTRRev	GTTGTCAACCATAACTGTAACACCTGCATCC	see Fw primer
T7-Erv25-ORFFw	TAATACGACTCACTATAGGGAAAGGATGCAGGTGTTAC	681
Erv25-ORFRev	GTGTGGTCCTCTTCCTTTGCTTAAATGATATG	see Fw primer
T7-Fcy1-3UTRFw	TAATACGACTCACTATAGGGATATTGGTGAGTAGAGC	520
Fcy1-3UTRRev	CCAATCCATGCCTATGGCCGCTATTTTCTTG	see Fw primer
T7-Fcy1-5UTRFw	TAATACGACTCACTATAGGGTTGTTCATCCCTTATCG	462
Fcy1-5UTRFw	GCCATTCCCCCTGTCACCATTAGCTATGA	see Fw primer
T7-Fcy1-ORFFw	TAATACGACTCACTATAGGGTAGCTAATGGTGACAG	512
Fcy1-ORFRev	CTGCGTGCTCTACTCACCAATATCTTCAAACC	see Fw primer
T7-Eno2-5UTRFw	TAATACGACTCACTATAGGGTCAGGAAGATGTTGGTTAC	897
Eno2-5UTRRev	GTGGAGGCACCAGATGGAACAATGGATC	see Fw primer
T7-Eno2-ORFFw	TAATACGACTCACTATAGGGACACCAAGCAACTAATAC	1377
Eno2-ORFRev	AAAGCACTTTACAACTTGTCACCGTGGTGGAAG	see Fw primer
T7-Nop53-GNN-Fw	TAATACGACTCACTATAGGGAGATGACAATGAAGAGGAAGAG	129
Nop53-GNN-Rev	TTCCTTATCAGATCCACTACATTGCGTACTTTC	see Fw primer
T7-Nop53-T7-ctrl.Fw	TAATACGACTCACTATAGGGCAGTCTTCAAGAAAGGGTAAG	147
Nop53-ctrl.Rev	TTTGCAAAGAGGTTATATCACTGGTACCATGCG	see Fw primer
T7-Ras2-ORF1Fw	TAATACGACTCACTATAGGGCATAAGAGAGTACAAGCTAG	309
T7-Ras2-ORF1Rev	GTAAGTCATAAGCTCATCAAGAGACGACTTGGACG	see Fw primer
T7-Ras2-ORF2Fw	TAATACGACTCACTATAGGGAAATGACAACTCCAAGC	436
Ras2-ORF2rev	AACTTATAATACAACAGCCACCCGATCCGCTCTTG	see Fw primer
T7-Snf5-Fw	TAATACGACTCACTATAGGGGATGATTCAGCAACGACACC	904
Snf5-Rev	GTTCGTACAACAAAGTATCCGT	see Fw primer

The following PCR primers were used to generate templates for *in vitro* transcription (T7 promoter is underlined). cDNA of HEK293 cells was used as template for PCR with Taq polymerase (GoTaq®, Promega Cat.# M3175).

T7-RASM_Fw	TAATACGACTCACTATAGGGACATACAAGCTGGTGGTG	600
RASM_Rev	CACATTGCAGTTTGTGGG	see Fw primer
T7-MYH4-Fw	TAATACGACTCACTATAGGGTGGCTGAGGAGCTGAAGAAG	217
MYH4-Rev	GGCCACAAGGAATTGAGTGA	see Fw primer
T7-FKTN_Fw	TAATACGACTCACTATAGGGTCTGCAAACTGGCCACTCAT	910
FKTN_Rev	CTGGATAACCTCATCCCAC	see Fw primer

The following oligonucleotide pairs (Fw/Rev) were annealed and used as direct templates for *in vitro* transcription (T7 promoter is underlined).

T7-(GATGAA)5-Fw	TAATACGACTCACTATAGGGATGAAGATGAAGATGAAGATGAAGATGAAG
(GATGAA)5-Rev	CTTCATCTTCATCTTCATCTTCATCTTCATCCCTATAGTGAGTCGTATTA
T7-(GATGCT)5-Fw	TAATACGACTCACTATAGGGATGCTGATGCTGATGCTGATGCTGATGCT
(GATGCT)5-Rev	AGCATCAGCATCAGCATCAGCATCAGCATCCCTATAGTGAGTCGTATTA
T7-(GATGTT)5-Fw	TAATACGACTCACTATAGGGGATGTTGATGTTGATGTTGATGTTGATGTT
(GATGTT)5-Rev	AACATCAACATCAACATCAACATCAACATCCCCTATAGTGAGTCGTATTA
T7-(GATTAA)5-Fw	TAATACGACTCACTATAGGGGATTAAGATTAAGATTAAGATTAAGATTAA
(GATTAA)5-Rev	TTAATCTTAATCTTAATCTTAATCTTAATCCCCTATAGTGAGTCGTATTA
T7-(GTTGTT)5_Fw	TAATACGACTCACTATAGGGGTTGTTGTTGTTGTTGTTGTTGTTGTTGTT
(GTTGTT)5_Rev	AACAACAACAACAACAACAACAACAACAACCCCTATAGTGAGTCGTATTA
T7-(GTGGTG)5_Fw	TAATACGACTCACTATAGGGGTGGTGGTGGTGGTGGTGGTGGTGGTGGTG
(GTGGTG)10_Rev	CACCACCACCACCACCACCACCACCACCACCCCTATAGTGAGTCGTATTA
T7-(GACGAC)5_Fw	TAATACGACTCACTATAGGGGACGACGACGACGACGACGACGACGACGAC
(GACGAC)5_Rev	GTCGTCGTCGTCGTCGTCGTCGTCGTCGTCCCCTATAGTGAGTCGTATTA
T7-stem2GAN-Fw	TAATACGACTCACTATAGGGGCTGCTGCTGCTGCTGATGAAGCTGCTGCTGCTGCT
Stem2GAN-Rev	AGCAGCAGCAGCAGCTTCATCAGCAGCAGCAGCAGCCCCTATAGTGAGTCGTATTA
T7-stem3GAN-Fw	TAATACGACTCACTATAGGGGCTGCTGCTGCTGCTGATGAAGATGCTGCTGCTGCTGCT
Stem3GAN-Rev	AGCAGCAGCAGCAGCATCTTCATCAGCAGCAGCAGCAGCCCCTATAGTGAGTCGTATTA
T7-stem4GAN-Fw	TAATACGACTCACTATAGGGGCTGCTGCTGCTGCTGATGAAGATGAAGCTGCTGCTGCTGCT
Stem4GAN-Rev	AGCAGCAGCAGCAGCTTCATCTTCATCAGCAGCAGCAGCAGCCCCTATAGTGAGTCGTATTA
T7-stem6GAN-Fw	TAATACGACTCACTATAGGGGCTGCTGCTGCTGCTGATGAAGATGAAGATGAAGCTGCTGCTGCTGCT
Stem6GAN-Rev	AGCAGCAGCAGCAGCTTCATCTTCATCTTCATCAGCAGCAGCAGCAGCCCCTATAGTGAGTCGTATTA
T7-stem8GAN-Fw	TAATACGACTCACTATAGGGGCTGCTGCTGCTGCTGATGAAGATGAAGATGAAGATGAAGCTGCTGCTGCTGCT
Stem8GAN-Rev	AGCAGCAGCAGCAGCTTCATCTTCATCTTCATCTTCATCAGCAGCAGCAGCAGCCCCTATAGTGAGTCGTATTA
T7-stem10GAN-Fw	TAATACGACTCACTATAGGGGCTGCTGCTGCTGCTGATGAAGATGAAGATGAAGATGAAGATGAAGCTGCTGCTGCTGCT
Stem10GAN-Rev	AGCAGCAGCAGCAGCTTCATCTTCATCTTCATCTTCATCTTCATCAGCAGCAGCAGCAGCCCCTATAGTGAGTCGTATTA

The following PCR primers were used to generate a Gateway (Invitrogen) compatible expression construct of the human Znf9 (Gateway overhangs in lower-case). cDNA of HeLa cells were used as template for PCR with KOD hot start Polymerase (Novagen Cat. # 71086).

Znf9-Fw	ggggacaagtttgtacaaaaaagcaggcttcATGAGCAGCAATGAGTGCTTCAAGTG
Znf9-Rev	ggggaccactttgtacaagaaagctgggtcGGCTGTAGCCTCAATTGTGCATTCC
