# Supplementary material for: Defining potentially conserved RNA regulons of homologous zinc-finger RNA-binding proteins
Source: Genome Biol. 2011 Jan 13;12(1):R3. doi: 10.1186/gb-2011-12-1-r3 (PMC3091301; doi:10.1186/gb-2011-12-1-r3)
Supplement: Additional file 5 — Distribution of ORFs with (GAN)7 repeats in Gis2p affinity isolates. The number of (GAN)7 motifs is plotted on the y-axis and ORFs are ranked on the x-axis based on decreasing SAM scores for Gis2p association. Range of genes with FDR <5% are marked with a bar. The numbers of (GAN)7 repeats as well as the number of genes with at least one (GAN)7 repeat in the ORF are indicated for Gis2p targets (red) and non-targets (green). [file gb-2011-12-1-r3-S5.PDF]

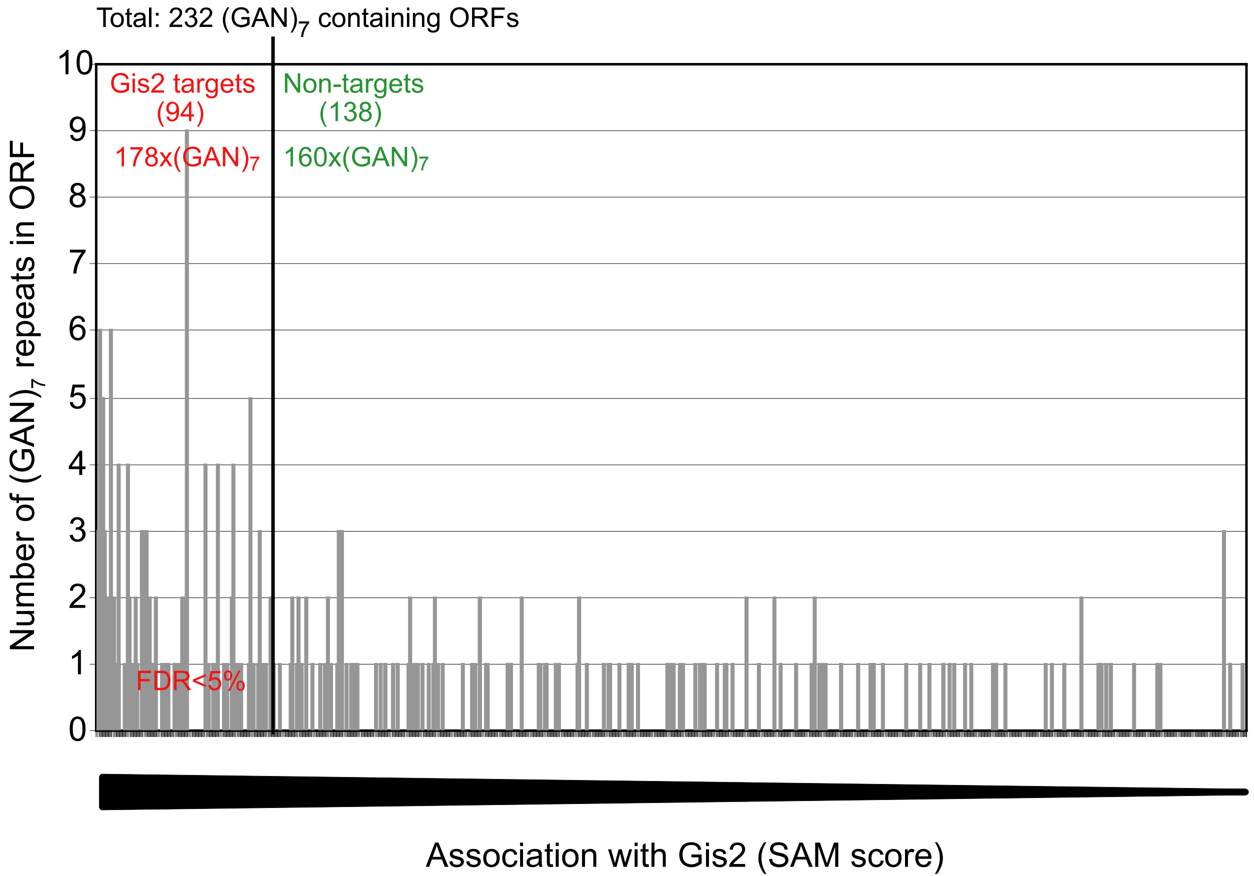

Total: 232 (GAN)7 containing ORFs
Gis2 targets (94)
178x(GAN)7
Non-targets (138)
160x(GAN)7
FDR<5%
Number of (GAN)7 repeats in ORF
0
1
2
3
4
5
6
7
8
9
10
Association with Gis2 (SAM score)
